# Strong stress-composition coupling in lithium alloy nanoparticles

**DOI:** 10.1038/s41467-019-11361-z

**Published:** 2019-07-31

**Authors:** Hyeon Kook Seo, Jae Yeol Park, Joon Ha Chang, Kyun Sung Dae, Myoung-Sub Noh, Sung-Soo Kim, Chong-Yun Kang, Kejie Zhao, Sangtae Kim, Jong Min Yuk

**Affiliations:** 1 Department of Materials Science and Engineering, Korea Advanced Institute of Science and Technology (KAIST), Daejeon, 34141 Republic of Korea; 2 KU-KIST Graduate School of Converging Science and Technology, Korea University, Seoul, 02841 Republic of Korea; 3 Center for Electronic Materials, Korea Institute of Science and Technology (KIST), Seoul, 02792 Republic of Korea; 4 Graduate School of Energy Science and Technology, Chungnam National University, Daejeon, 34134 Republic of Korea; 5 School of Mechanical Engineering, Purdue University, West Lafayette, 47907 IN USA

**Keywords:** Nanoparticles, Metals and alloys, Transmission electron microscopy, Batteries

## Abstract

The stress inevitably imposed during electrochemical reactions is expected to fundamentally affect the electrochemistry, phase behavior and morphology of electrodes in service. Here, we show a strong stress-composition coupling in lithium binary alloys during the lithiation of tin-tin oxide core-shell nanoparticles. Using in situ graphene liquid cell electron microscopy imaging, we visualise the generation of a non-uniform composition field in the nanoparticles during lithiation. Stress models based on density functional theory calculations show that the composition gradient is proportional to the applied stress. Based on this coupling, we demonstrate that we can directionally control the lithium distribution by applying different stresses to lithium alloy materials. Our results provide insights into stress-lithium electrochemistry coupling at the nanoscale and suggest potential applications of lithium alloy nanoparticles.

## Abstract (web summary)

Electrochemical reactions can generate stresses that detrimentally affect battery electrodes. Here, the authors directly image the lithiation of core-shell tin-based nanoparticles and show that lithium can be redistributed by applying stress and change the nanoparticle composition.

## Introduction

Mechanical stress critically affects various chemical processes ranging from electrochemical energy storage^1,2^ to catalytic reactions^3,4^ to polymerization^5^. In lithium (Li) insertion electrochemistry, the large stress evolution during lithiation cycles poses major challenges to incorporating alloying anodes in commercialized batteries^6,7^. In addition to causing electrode cracking, stress couples to the insertion electrochemistry, affecting the electrode potential, lithiation kinetics, and Li composition^8,9^. These electrochemistry–stress couplings have been studied in depth, including the stress contribution to chemical potential^10^ or stress-induced kinetic retardation^11,12^. However, how stress solely changes the Li composition in the nanoscale has not been unexplored experimentally^13^, because stress, diffusion, and Li composition are mutually coupled to one another. Understanding the stress–composition coupling would potentially open up various technological applications such as energy harvesters, while providing scientific insights into stress–matter interaction.

While directly observing the composition change under applied stress may shed light on this fundamental question, experimentation has thus far remained difficult^1,14^. To observe the role of stress in battery electrode materials, it is necessary to stress the active material within the iso-chemical potential reservoir (the electrolyte) and simultaneously observe the compositional and morphological changes in situ. Since the compositional spatiodynamics in battery materials occurs on a nanoscale, nanoscale observations are also essential^15^. Realizing this capability has been challenging with the current state-of-the-art characterization techniques such as in situ transmission electron microscopy (TEM) on an encapsulated liquid^16^.

Thus, here, we use in situ graphene liquid cell electron microscopy (in situ GLC-EM)^17,18^ to visibly track the temporal phase and morphological evolution of a single nanoparticle while it lithiates under stress. To apply stress in situ, we use tin-tin oxide (Sn-SnO_2_) core-shell nanoparticles. During lithiation, the oxide shell acts as an effective instrument that applies large hydrostatic compression to the core, while undergoing tensile hoop stress itself. The constant electron irradiation from the TEM column drives the lithiation of the nanoparticles immersed in the liquid specimen, which is a condition similar to the galvanostatic lithiation in a battery cell. Sn in tetragonal phase (β-Sn, space group *I*4_1_/*amd* (141), *a* = 5.83 Å, *c* = 3.18 Å) is chosen for its fast Li diffusivity^19^, in efforts to minimize the effect of stress-driven kinetic retardation. Also, owing to its relatively low alloying energy of −0.48 eV, Li_3.5_Sn is a suitable material for studying the interaction between electrochemistry and stress, which occurs on a comparable energy scale.

## Results

### Abnormal phase evolution during shelled particle lithiation

**Fig. 1:**
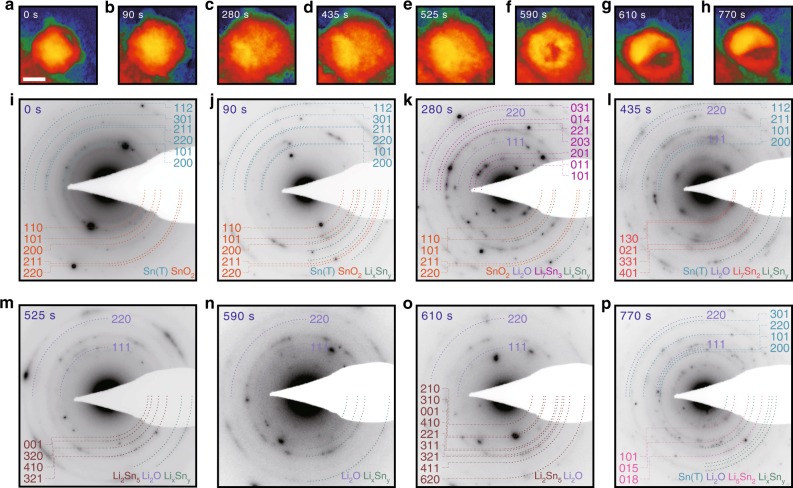
Lithium (Li)-deficient phases nucleated during lithiation under stress. **a**–**h** Lithiation-induced volume changes during in situ lithiation of a Sn-SnO_2_ core-shell particle. **i**–**p** The electron diffraction patterns taken at the respective (**a**–**h**) moments. The still snapshots are captured from Supplementary Movie 1. The scale bar indicates 100 nm

Figure 1 and Supplementary Movie 1 track the phase evolution of a Sn-SnO_2_ core-shell nanoparticle during in situ lithiation. The setup for GLC-EM and the lithiation condition inside GLC-EM are described in the Supplementary information (Supplementary Fig. 1, Supplementary Table 1, and Supplementary Notes 1 and 2). The electron diffraction patterns (EDPs) in Fig. 1 reveal that the core-shell nanoparticle lithiates by subsequently nucleating partially lithiated Sn (Li_*x*_Sn_*y*_) at 90 s (Fig. 1b, j), crystalline Li_7_Sn_3_ at 280 s (Fig. 1c, k), and Li_7_Sn_2_ phase at 435 s (Fig. 1d, l). We observe that the EDPs from these phases appear in order, as well as those from Sn core, SnO_2_ shell, and Li_2_O generated by SnO_2_ shell conversion (SnO_2_ + 4Li → Sn + 2Li_2_O)^20^. The SnO_2_ peaks do not disappear until 280 s, indicating that SnO_2_ partially remains until Sn is heavily lithiated. This is surprising since the calculated average potential for SnO_2_ shell conversion (1.5 V) is notably higher than that of Sn (0.6 V) and suggests kinetically limited SnO_2_ lithiation^21^. The Sn core could be lithiated first by Li migration through grain boundaries in the SnO_2_ shell^22^.

Further lithiation, involving significant shell-imposed stress on the core, exhibits unanticipated phase evolution. Instead of forming a fully lithiated Li_22_Sn_5_ phase, the observed Li_7_Sn_2_ phase at 435 s evolves into Li_*x*_Sn_*y*_ or Li_2_Sn_5_ phases (Fig. 1e–g, m,o). These Li-deficient phases are consistently observed, and the final phases observed after 770 s of lithiation are Sn, Li_5_Sn_2_, Li_*x*_Sn_*y*_, and Li_2_O (Fig. 1h, p and Supplementary Table 2). The intermediate phases and even pristine Sn that disappeared during initial lithiation remarkably reappear with the continued lithiation.

These phenomena are consistently observed during galvanostatic lithiation of similar particles in conventional battery cells. For example, in situ small-angle X-ray scattering (SAXS) experiments during lithiation show a sudden increase in Sn content near Li_2_Sn nominal composition (Supplementary Fig. 2). The ex situ X-ray diffraction (XRD) patterns of the fully lithiated core-shell nanoparticles reveal largely Li-deficient phases (Supplementary Fig. 3 and Supplementary Note 3)^23^. During the lithiation of bare Sn particles without oxide shells, however, the Li-deficient phases do not appear. These particles fully lithiate into the Li_22_Sn_5_ phase in the identical GLC setup, clearly indicating the role of the oxide shell (Supplementary Fig. 4 and Supplementary Movie 2).

### In situ observation of Kirkendall voiding during lithiation

**Fig. 2:**
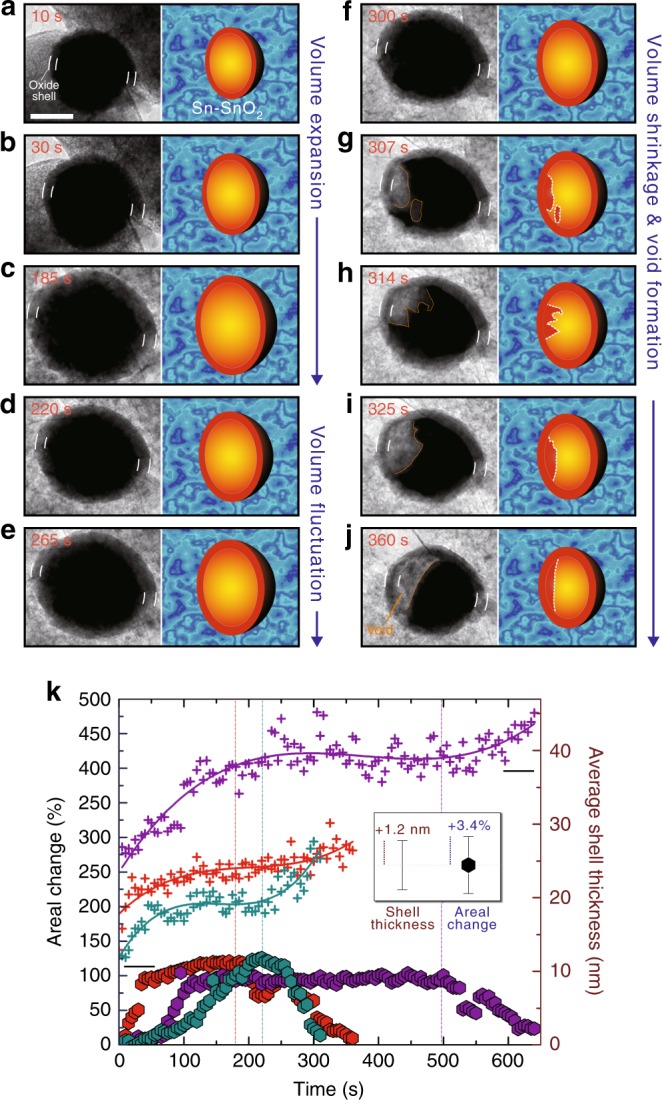
Void-forming morphological evolution during lithiation under stress. **a–j** Time-series transmission electron microscopy (TEM) images from Supplementary Movie 3 showing dimensional and morphological evolutions of a Sn-SnO_2_ nanoparticle during in situ lithiation for 360 s. White borderlines on each panel note the oxide shell edges. Orange curves highlight the fluctuating void surface on the Sn core. The illustrations next to the TEM images describe the morphological evolution of the particles, including swelling, shrinking, and void formation/evolution. The scale bar indicates 100 nm. **k** The areal changes (hexagons) and average oxide shell thicknesses (cross-shapes) plotted over the lithiation time, for three similar Sn-SnO_2_ particles under two different electron-beam conditions. The error bar indicates the standard deviations obtained from 10 image frames separated by 0.5 s

The abnormal lithiation behavior under stress also accompanies unique morphological evolution, as shown in the time-series TEM images (Fig. 2 and Supplementary Movie 3). The nanoparticle expands upon lithiation and reaches maximum areal expansion of 120% at 185 s (Fig. 2a–c, Supplementary Table 3, and Supplementary Notes 4 and 5). As lithiation continues, the nanoparticle briefly fluctuates in size (Fig. 2d, e), followed by spontaneous volume shrinkage and Kirkendall void nucleation inside the core (Fig. 2f, g). The core then wobbles like a liquid, forming a void and a single meniscus at 360 s^21,24–26^. The core’s areal expansion at this point is only 8%, suggesting a large amount of Li extraction from the core. Indeed, the volume expansion analyses show that the composition in the core changes from Li_3.5_Sn to Li_0.4_Sn^27,28^, corresponding closely to the reappearance of Li-deficient phases in Fig. 1. Such voided morphology also consistently appears in the ex situ observations of galvanostatically cycled particles in the conventional battery setup (Supplementary Figs. 5–7).

The detailed morphology analyses reveal where Li migrates during the core dealloying. Figure 2k plots the areal change of the cores and average shell thicknesses during lithiation for three different core-shell particles (Supplementary Movies 1, 3, and 4 and Supplementary Figs. 8–10). The shell thicknesses increase during the initial lithiation and soon reach plateaus^22,29^. The stalled shell thickening, however, shows a notable increase as the cores begin shrinking. This negative correlation between the core areas and shell thicknesses suggests that Li migrates from the core into the shell.

**Fig. 3:**
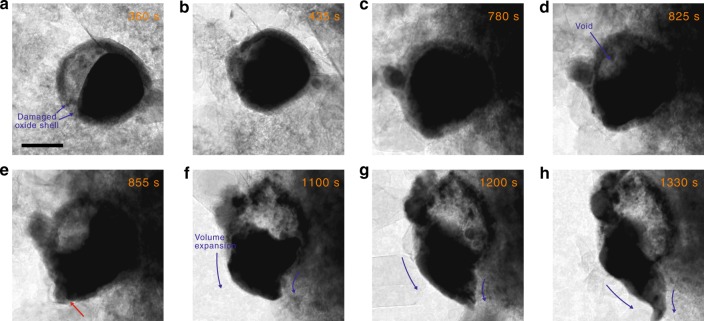
Time-series images of continued lithiation of a voided particle. **a**–**c** The particle repeats lithiation once shell-induced mechanical interaction is released and **d** self-discharge again upon sufficient lithiation. **e**–**h** As the shell tears apart, however, the core continues normal lithiation despite being partially attached to the shell. The scale bar indicates 100 nm

Allowing the once voided particle to further evolve under constant electron-beam irradiation reveals repeated void-forming behavior (Supplementary Movie 5). The particle undergoes further lithiation, filling in the void dynamically formed during core dealloying (Fig. 3a–d). The continued lithiation shows that once the shell-induced mechanical constraint is removed, the core undergoes normal lithiation again. After sufficient expansion and complete filling of the void, the lithiated core repeats the shrinkage and dealloying, nucleating a void in a similar manner (Fig. 3d, e). At this point, the repeated mechanical interaction partially breaks the shell apart, freeing the core from mechanical constraint. The lithiation of the core thereafter continues smoothly without any shrinkage or the formation of voids for an extended time (475 s, Fig. 3e–h), similar to our observations of oxide-free Sn nanoparticles (Supplementary Fig. 4). The core expansion is directed towards the region where the shell is partially torn apart (indicated with a red arrow in Fig. 3e), possibly due to the fast Li transport at this region compared to sluggish Li transport through the lithiated oxide. It is noted that the core is still in contact with the oxide shell, although not completely covered by the shell. This strongly suggests that the core’s dealloying is observed only when the shell completely covers the core surface, that is, when the shell imposes a mechanical constraint on the core. This also directly shows that the dealloying of the core is not driven by the chemical potential difference between the lithiated shell and the core.

### Stress–composition coupling in Li alloys

**Fig. 4:**
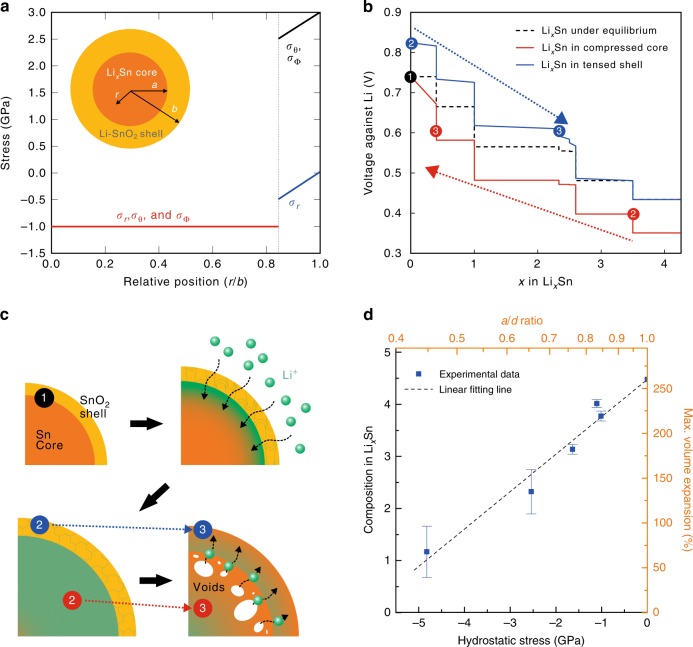
Thermodynamic rationale for the strong stress–composition coupling. **a** The stress distribution within the particle during lithiation according to the relative radial position from the particle center, with the inset showing the lithiated particle geometry. The shell undergoes tensile plastic deformation while imposing compressive hydrostatic stress of 1 GPa on the core. **b**, **c** The stress effect on the equilibrium potential of the Li-Sn alloy system (**b**) and schematic illustrating the spontaneous core dealloying based on the stress-driven potential differences (**c**). The numbered circles illustrate the discrete timeframes during lithiation. **d** The experimentally observed stress–composition coupling in Li_*x*_Sn. The maximum volume expansion observed before dealloying initiation according to the core-to-particle radii ratio (*a*/*b*). The error bars indicate standard deviations in volume from 10 image frames separated by 1 s

The observed phase and morphological evolution bring our attention to their thermodynamic rationale. Figure 4 illustrates the stress distribution inside the core-shell nanoparticle during lithiation and its effect on the composition. Lithiation-induced volume expansion imposes tensile hoop stress on the shell while imposing compressive stress on the core (Fig. 4a, Supplementary Fig. 11, Supplementary Table 4, and Supplementary Notes 6, 7)^30,31^. This stress contributes to Sn’s equilibrium discharge voltage (Supplementary Fig. 12 and Supplementary Note 8)^10^. As particles lithiate from point 1 (Fig. 4b), the shell-imposed stress lowers the core’s potential in proportion to the amount of stress (red point 2 in Fig. 4b). At the same time, the Sn islands in the lithiated oxide shell undergo tensile hoop stress and experience elevated potential (blue point 2 in Fig. 4b). The potential difference between the core and the shell drives Li migration from the core into the shell, causing Kirkendall voids in the former (Supplementary Fig. 13 and Supplementary Note 9). The migration continues until isopotential is reached at around 0.6 V against Li (red and blue points 3 in Fig. 4b), where the phases approximately match with the final phases observed in Fig. 1. This whole process is illustrated schematically in Fig. 4c.

The model also suggests that increased stress leads to reduced Li composition. Specifically, thick shells induce a large potential difference by stress and thus result in dealloying at earlier stages of lithiation (Supplementary Figs. 14 and 15, Supplementary Note 10, and Supplementary Movie 6). Performing identical experiments with a small core-to-particle radii ratio (*a*/*b* ratio) indeed results in a reduced maximum Li composition compared to that with the thin shell (Supplementary Fig. 16). Figure 4d shows a remarkable correlation between the *a*/*b* ratio and the maximum volume expansion observed. Interestingly, since the applied hydrostatic stress and volume expansion scale with log (*a*/*b*) and the Li composition, respectively, the achievable Li composition is proportional to the applied stress.

## Discussion

Considering that the energy scales of electrochemical and mechanical phenomena are in general considered different, the stress-driven core dealloying is surprising. It is noted, however, that the free energy of formation (equivalent to the electrochemical voltage at which the compound forms) of Li_3.5_Sn is only −0.48 eV. In fact, most lithium binary alloys form well under 1 V against Li/Li^+^, with 0.74 V for Li_2_Sn_5_ being the highest among all lithium tin binary alloys. This energy scale is much smaller than the level at which typical Li-ion batteries with Co^3+/4+^ redox couples operate (~4 V) since no d-band-based redox couples come into play, but only alloy thermodynamics of the Li-Sn system. This energy scale is in fact similar to the energy scales of constraint-induced stress modeled in this work. Supplementary Fig. 14 shows the potential contribution from the mechanical interaction on the shell and the core, assuming a plastically deforming oxide shell. This potential contribution reaches 0.4 V when the core-to-particle radii ratio falls below 0.8.

In alloy thermodynamics, stress is well known to affect the alloy composition and phase behavior. During creep tests at 1223 K, nickel-based single crystal SC16 alloy has been reported to form Cr-deficient and Ni-rich γ phases due to creep stresses^32^. In eutectoid Zn-Al alloys, metastable η′ phases are reported to undergo a phase transformation due to creep^33^. Since Li possesses high diffusivity at room temperature, the stress-induced Li composition change and phase transformations can be viewed as a room temperature creep. In this sense, the observations in this work are well aligned with the classical creep phenomena in metallurgy. It is noted that exciting behaviors in stress-induced delithiation on a nanoscale have already been reported and demonstrated. By coating spherical Si particle surfaces with polypyrrole, Luo et al.^34^ observed that an amorphous Li_*x*_Si portion within the nanoparticles self-discharged into amorphous Si^34^. It has also been reported that the constraint-induced mechanical interaction can be a significant factor impeding electrochemical lithiation of silicon^30^.

One may argue that the void formation is in fact phase separation behavior of lithiated tin into Li-rich and Li-poor phases. We note that the elemental mapping of a voided particle exhibits uniform Li distribution and segregated Sn within the particle (Supplementary Fig. 7). The uniform Li distribution first rules out the possibility of the void being a Li-rich phase formed via phase separation. The thickness ratio obtained via electron energy loss spectroscopy (EELS) (Supplementary Fig. 7f, g) further show that the voided area exhibits a sudden decrease in thickness with only approximately 1/3 that of the dealloyed core. The thickness likely corresponds to the outer shell, and strongly asserts for the void formation. The shell, however, does not exhibit notably increased Li concentration. This may be due to the fact that two-dimensional (2D) elemental mapping is difficult to deconvolute the signals from thick and thin regions; similar Li concentration from considerably thinner shell suggests increased Li concentration within the shell than that from the core.

It is noted that the stress model developed above only depends on the *a*/*b* ratio, and not on the absolute size of the particle. However, the model also assumes that the shell maintains its constraint against the core, despite the core expansion. Such mechanical integrity can be expected only from nanoscale cores and shells, as large particles may be subject to size-dependent degradation mechanisms. Over the past ten years, we learned that materials become stronger^35–37^ as the material approaches sub-microscale. Our observations occur at a scale of 100 nm, where the shell and core are expected to possess nearly ideal strengths.

The stress–composition coupling implies that we can deliberately redistribute Li according to the applied stress. We demonstrate this by fabricating an electrochemically driven mechanical energy harvester with Li_3_Sn thin films^2^ (Supplementary Fig. 17). The measured short-circuit current corresponds to the amount of redistributed Li between the identical electrodes due to the different stresses applied by bending. Unbending the device does not generate an electric current in the opposite direction since the two thin films in two-phase equilibria (between Li_5_Sn_2_, Li_7_Sn_2_) maintain a fixed Li chemical potential over a given composition range. This indicates that the current generated is entirely due to the stress gradient and not due to the chemical composition difference. Along with the reported Li_*x*_Si-based harvesters, the present findings suggest that stress–composition coupling is general in Li binary alloys, although the degree of its effect may be modulated by the stress-driven kinetic retardation^11^.

One may also question whether irradiating a liquid cell with an electron beam may cause unexpected side effects. We also seriously considered the similarity and differences between our lithiation technique and the electrochemical conditions. The critical question here is whether the stress–composition coupling we observe is caused by side effects of the electron beam. To ensure scientific rigorousness, we employed multiple characterization techniques including ex situ TEM analyses and in situ SAXS during galvanostatic lithiation. These analyses show the Li-deficient phases and voided particle morphology, confirming that the results are reproducible under galvanostatic conditions. In addition, the sharp contrast between monotonic lithiation of oxide-free Sn nanoparticles and the abnormal lithiation of core-shell nanoparticles under identical GLC condition indicates that stress-driven Li migration is not caused by electron-beam irradiation.

Despite the foregoing, we employed a commercially available electrolyte solution (1 M lithium hexafluorophosphate (LiPF_6_) salt in ethylene carbonate/diethyl carbonate (EC/DEC) mixture) known to be relatively stable against electron-beam irradiation^38^, in efforts to minimize possible electrolyte decomposition (both salt and solvent). We also expected that the conductive graphene layers covering the electrolyte would provide additional protection by radiolytic damage relaxation^17,18^. Under these conditions, control experiments confirm that no visible electrolyte degradation occurs under 7.71 × 10^−14^ A nm^−2^ electron-beam irradiation, the beam condition employed for the in situ lithiation experiments (Supplementary Fig. 17). The time-series bright-field TEM images show natural liquid fluctuations, yet with no gaseous bubble formation or decomposed particle precipitation.

High-intensity X-ray-based in situ experimental techniques that do not involve any electron-beam damages have recently been developed^15,39^. Nonetheless, these techniques visualize the redox-active transition metal species and may not yet be applied to lithium alloys without transition metal species. In addition, the complex phase and morphological evolution during lithiation of core-shell nanoparticles require nanoscale resolution currently only capable with TEM analyses.

Despite the powerful capabilities of GLC-EM, caution should be exercised because the damage from the electron beam has been reported to be more severe in water, as compared to electrolyte solutions. Schneider et al.^40^ recently showed that a large electron-beam dose might deactivate the etching chemistry of a water-based solution and erroneously show growth behavior of nanoparticles. The degree of radical formation yield under irradiation has been quantified as *G* values, and Abellan et al.^41^ showed that the *G* values for aqueous electrons (e_aq_^−^) are approximately 20 times higher in magnitude than those of H_2_ radical formation in toluene. For this reason, electron-beam irradiation to drive redox reactions in organic solvent-based electrolytes has been demonstrated extensively as a successful experimental technique^18,42–44^, while those for aqueous chemistry has been more challenging.

In summary, we directly observe the stress–composition coupling in Li alloy nanoparticles via GLC-EM. Lithiating a Sn-SnO_2_ core-shell nanoparticle results in substantive stress development within the particle, and constraint-induced mechanical stress results in a significant Li composition change among lithiated core-shell nanoparticles. The strong stress–composition coupling demonstrated in this work has general implications for energy harvesters, sensors, and battery electrode designs. The ability to redistribute a large amount of Li by applying stress implies immediate applications for mechanical energy harvesters or storage systems. Also, by promoting the coupling effect via engineering the Li diffusivity, we can toughen the electrodes to alleviate their fracture or pulverization during lithiation/delithiation cycles. For instance, the tensile stress field near crack tips will invite nearby Li to fill in and blunt the crack tip, retarding crack propagation^7^. In battery electrode designs, stress can be an active design variable controlling the maximally achievable Li composition while at the same time preventing electrode fracture.

## Methods

### Materials and GLC preparation

Commercially available Sn (β-phase) nanoparticles with a diameter range of 50–200 nm from Sigma-Aldrich were used. The Sn-SnO_2_ core-shell structure was synthesized by thermal oxidation of the Sn particles under an oxygen environment at 120 °C for 24 h. The particle maintains strong crystallinity in both its core and shell (Supplementary Fig. 18). Graphene was synthesized on a copper substrate (0.025-mm-thick foil, Alfa Aesar) by chemical vapor deposition (Supplementary Fig. 19, Thermal CVD System, Scien Tech Inc.). The Li-ion battery electrolyte is 1 M LiPF_6_ salt dissolved in ECs and DEC solution mixed at a 1:1 volume ratio.

The GLC is directly fabricated by drop-casting 20 μL of Sn nanoparticle-dispersed electrolyte solution on two overlapped graphene-coated TEM grids (Au 300 mesh, quantifoil R2/2, SPI supplies), followed by suctioning the extra solution^17,18^. By van der Waals attractions between the graphene sheets, a few micron-sized liquid electrolyte pockets containing Sn nanoparticles are formed. After vacuum drying for 24 h, the sample was loaded into a TEM column for in situ experiments. All liquid cell fabrications were conducted in a high-purity argon (Ar)-filled glove box, with moisture (H_2_O) and oxygen (O_2_) concentrations <1 ppm.

### Electrochemical cell tests

For electrochemical tests, Sn working electrodes were prepared by the slurry casting method. The slurry was composed of Sn nanoparticles, carbon black (acetylene, Alfa Aesar), and polyvinylidene fluoride (PVDF, Sigma Aldrich) uniformly mixed at an 8:1:1 mass ratio in a 1-methyl-2-pyrrolidone (Sigma-Aldrich) solution. After depositing the slurry onto a Cu current collector by doctor blading, we dried the slurry for 12 h in a vacuum chamber (OV-12, Jeio Tech). A half-cell (ECC-STD, EL-CELL) was assembled with the slurry-based working electrode, Li metal counter electrode, and separator in an argon-filled glove box. LiPF_6_-based electrolytes were used for the cell assembly, which had an identical composition to that used in the GLC fabrication above. With a cell tester (PARSTAT MC 1000, Princeton Applied Research), charging and discharging measurements were conducted under a constant current condition of a 0.1 C-rate at room temperature. The cutoff voltages were 0.05 and 3.0 V. Galvanostatic intermittent titration technique measurements were carried out for both pristine Sn and core-shell Sn-SnO_2_ nanoparticles by periodically applying 1-h duration current pulses at 0.05  C, with 5-h relaxation after each pulse (Supplementary Note 8). The open-circuit voltage at a specific composition was defined by the recovered Li potential after 5-h rest time and is plotted as a function of the Li composition in Supplementary Fig. 20.

### In situ and ex situ TEM characterizations

A JEM-3010 (300 kV, JEOL) microscope with a high-speed charge-coupled device camera (SC200, Gatan) was used to observe the real-time lithiation dynamics of Sn nanoparticles. Supplementary Movies showing the morphological evolutions were recorded with the Gatan Digital Micrograph software (recording rate: 3 frames s^−1^, Gatan Microscopy Suite). The electron-beam dosage was maintained in a range of 3.0–8.0 A cm^−2^ to initiate the lithiation as well as for TEM imaging. Ex situ TEM observations were carried out with a Tecnai G^2^ F30 S-Twin microscope (300 kV, FEI). After cycling the electrochemical cells, we disassembled the cells under an inert Ar atmosphere. Residual electrolyte on the Sn electrode surfaces was removed by rinsing and sonicating the surface several times with a dimethyl carbonate (DMC) solution. The Sn-dispersed DMC solution was then drop-casted onto a perforated carbon-coated TEM grid (Cu, 300 Mesh, SPI Supplies). TEM imaging and energy-dispersive spectroscopy elemental analyses were conducted after drying the solvent at room temperature for 12 h. EELS spectra are obtained at 300 kV accelerating voltage (Supplementary Fig. 21 and Supplementary Note 2)

### **In situ** SAXS measurements

The crystalline phase evolution during lithiation of Sn-SnO_2_ particles was tracked via SAXS (D/MAX-2500 with R-AXIS IV++ diffractometer, Rigaku) (Supplementary Fig. 3). The X-ray beam (Cu K_α_) was generated by electron-beam irradiation on a rotating anode type Cu plate at 50 kV and 100 mA. The X-ray beam filtered with a 0.15-mm-diameter slit was collimated into the assembled coin cell. To allow both the incident and scattered beam to pass through the lithiated Sn-SnO_2_ particle electrode, both sides of the coin cell case have X-ray transparent polymer windows. Supplementary Fig. 22 schematically illustrates the configuration of our in situ SAXS experiment setup and shows the actual device setup. For the electrochemical cycling test, the coin cell was assembled with a Li foil counter electrode and discharged under a constant current condition (0.05 C). The data acquisition was carried out at 1-h intervals. Using Rigaku-Display software, we processed the obtained time-series 2D intensity maps into time-series 1D spectra by summing the intensity for all radial directions as a function of the scattering angle, ranging from 15° to 50°.

### Ex situ XRD measurements

A high-resolution powder X-ray diffractometer (SmartLab, 45 kV 200 mA, Rigaku) was used for crystalline phase analyses in the cycled Sn-SnO_2_ nanoparticles. After cycling, we disassembled the coin cell in an Ar-filled glove box and scraped the active materials off a Cu current collector. The active materials were then loaded into a home-made air-tight holder with an X-ray transparent Kapton polyimide (PI) film window, avoiding air exposure during measurements. Under the normal *θ*/2*θ* scan mode, the 2*θ* scan range, scan speed, and step size were set to be 20–35°, 4° min^−1^, and 0.01°, respectively.

### Ab initio calculations

All density functional theory calculations were performed using Vienna ab initio simulations package^45^. The exchange correlation functional was treated via generalized-gradient approximation^46^ and projector-augmented wave method implemented with Perdew–Burke–Erzenhof functional were used^47^. Both ionic positions and cell parameters were allowed to fully relax during the calculations, and an energy cutoff of 520 eV was used. K-point grid density of at least 1000 per atom was used, and spin-polarized calculations were performed with no Hubbard-like U correction.

### Energy harvester fabrication

The energy harvesters were fabricated on 25 μm PI substrates, with 100- and 15-nm-thick current collector layers of Pt and Ti, respectively. Sn electrodes were then deposited up to 100 nm, at a deposition rate of 0.2 Å s^−1^. All depositions were performed by sputtering target metals onto the substrates. The Sn thin films were electrochemically lithiated up to Li_3_Sn composition and were assembled into an energy harvester by cutting the thin film into half, sandwiching a layer of commercial Li electrolyte (EC:DEC 1:1 volume ratio, 1 M LiPF_6_), and packaging with an Al-covered pouch cell. The short-circuit current was measured with a Keithley 6485 picoammeter, while the device was being bent via an Arduino-controlled servo motor with 8 mm radius of curvature.

## Supplementary information

Supplementary Information

Supplementary Movie 1

Supplementary Movie 2

Supplementary Movie 3

Supplementary Movie 4

Supplementary Movie 5

Supplementary Movie 6

Description of Additional Supplementary Files

## Data Availability

All data supporting the conclusions of this study are included in the manuscript and its Supplementary materials.
