# Supplementary material for: Strong stress-composition coupling in lithium alloy nanoparticles
Source: Nat Commun. 2019 Jul 31;10:3428. doi: 10.1038/s41467-019-11361-z (PMC6668403; doi:10.1038/s41467-019-11361-z)
Supplement: Supplementary file 1 — Supplementary Information [file 41467_2019_11361_MOESM1_ESM.pdf]

Strong Stress-Composition Coupling in Lithium Alloy Nanoparticles

Supplementary Information

Seo *et al*.

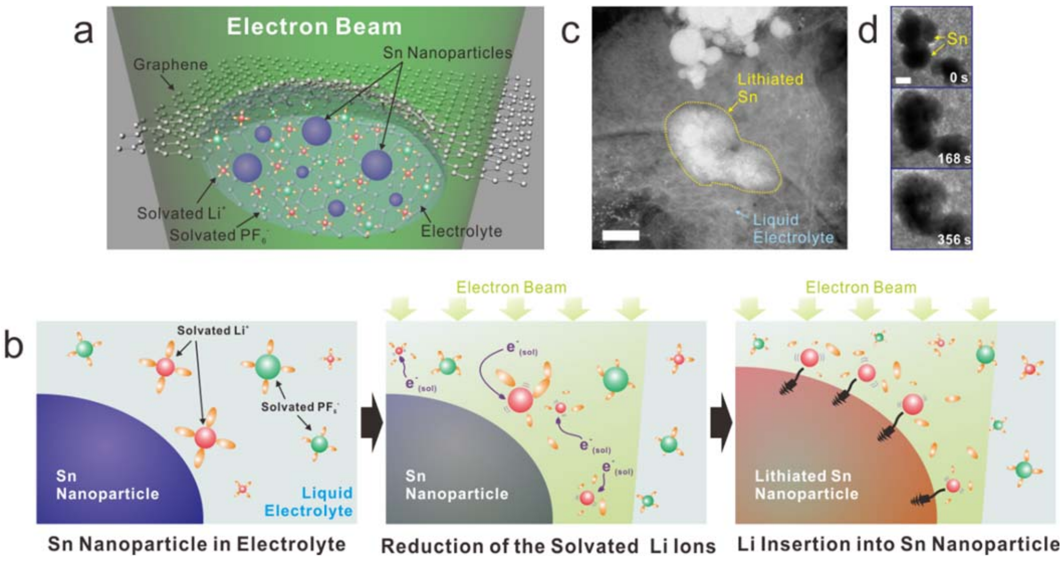

Supplementary Figure 1. GLC-EM employed to observe lithiation in commercial electrolyte bubbles. a A schematic describing the GLC-EM method. Commercial Li-ion electrolyte with Sn nanoparticles is encapsulated between two graphene sheets. **b** The schematics show the lithiation mechanism upon electron irradiation. Constant electron beam current irradiated on the liquid specimen rapidly saturates the reactive solvated electrons ($e^{-}_{(sol)}$), which drives the lithiation reaction. The solvated electrons separate the solvent molecules on the solvated Li^{+} ions. The Li^{+} ions migrate to nearby Sn nanoparticles as the ions get reduced by the solvated electrons ($xLi^{+} + xe^{-}_{(sol)} + Sn \rightarrow xLi + Sn \rightarrow Li_xSn$). **c** The STEM image shows the fabricated graphene cell with lithiated Sn nanoparticles inside liquid electrolyte. The lithiated Sn nanoparticles are outlined with a yellow dashed line. **d** Time-series BF-TEM images display the lithiation process. The scale bars in **c** and **d** indicate 200 and 100 nm, respectively.

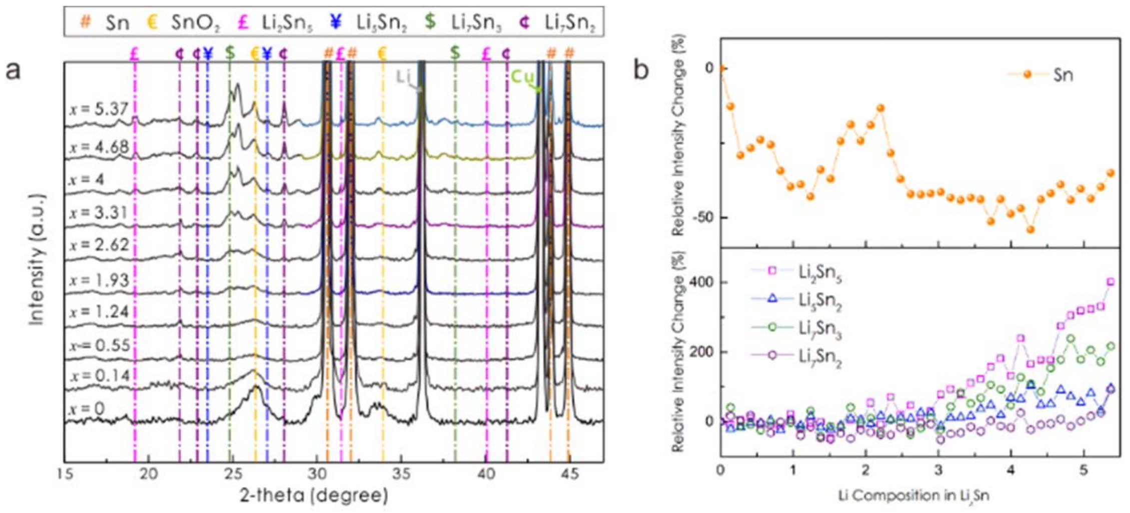

Supplementary Figure 2. *In situ* X-ray scattering reveals quantitative phase evolution during electrochemical lithiation of Sn-SnO$_2$ nanoparticles. a A series of X-ray scattering spectra taken by *in situ* SAXS measurement ranging from 15 to 48º. The *x* value of each spectrum means calculated Li composition in Li$_x$Sn alloy. **b** Normalized intensity variations with Li content for the crystalline phases shown in **a**. The intensity of Cu (111) is chosen as a reference for normalization. Sn peak intensity fluctuates at the initial lithiation stages, while the other Li-Sn alloy phase gradually increase over the course of lithiation, especially after Li$_3$Sn.

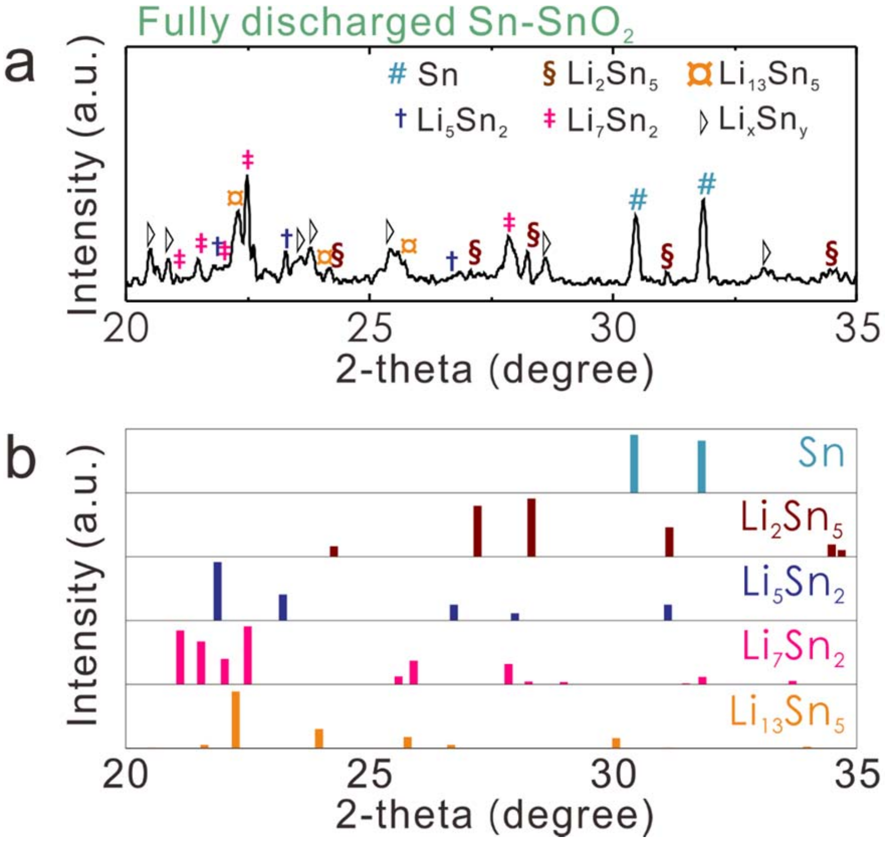

Supplementary Figure 3. The mixed phases observed in galvanostatically lithiated Li_xSn particles. a *Ex situ* XRD results obtained from fully discharged Sn-SnO_2 nanoparticles. **b** The reference peak positions of β-Sn and Li-Sn intermetallics identified from the Inorganic Crystal Structure Database (ICSD) (Supplementary Table 1).

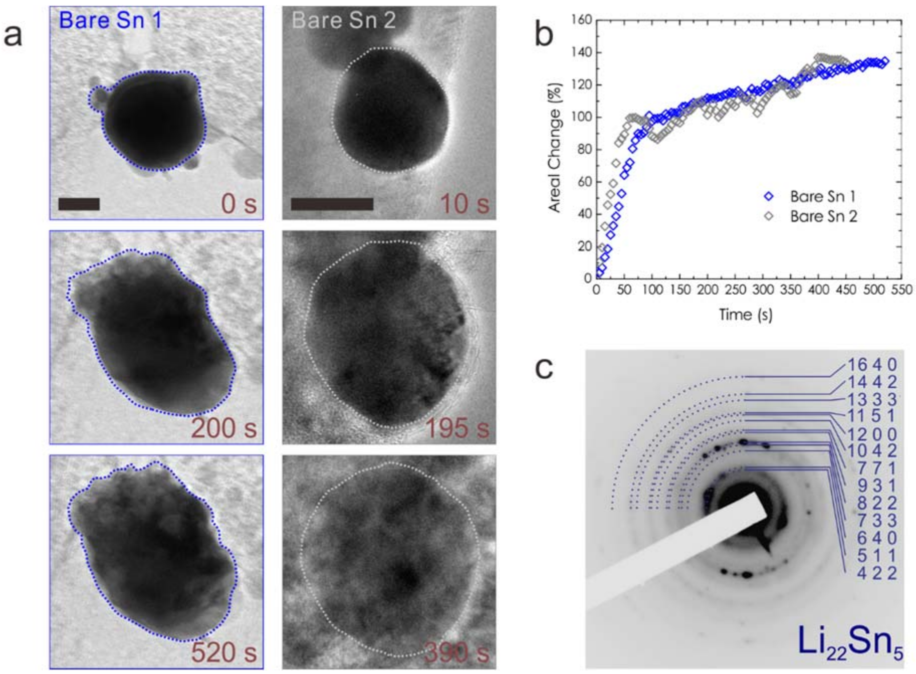

Supplementary Figure 4. Complete lithiation of pristine Sn nanoparticles. a Time-series TEM images show the morphological evolutions of two bare Sn particles (labeled as Bare Sn 1 and 2). **b** The measured areal changes of Bare Sn 1 and 2 with respect to the lithiation time reveals continuous lithiation for the two particles. We notice two-stage lithiation kinetics. **c** The electron diffraction patterns on Bare Sn 1 after 520 s of continued lithiation shows the formation of $Li_{22}Sn_5$ phase. The scale bars in **a** indicate 100 nm.

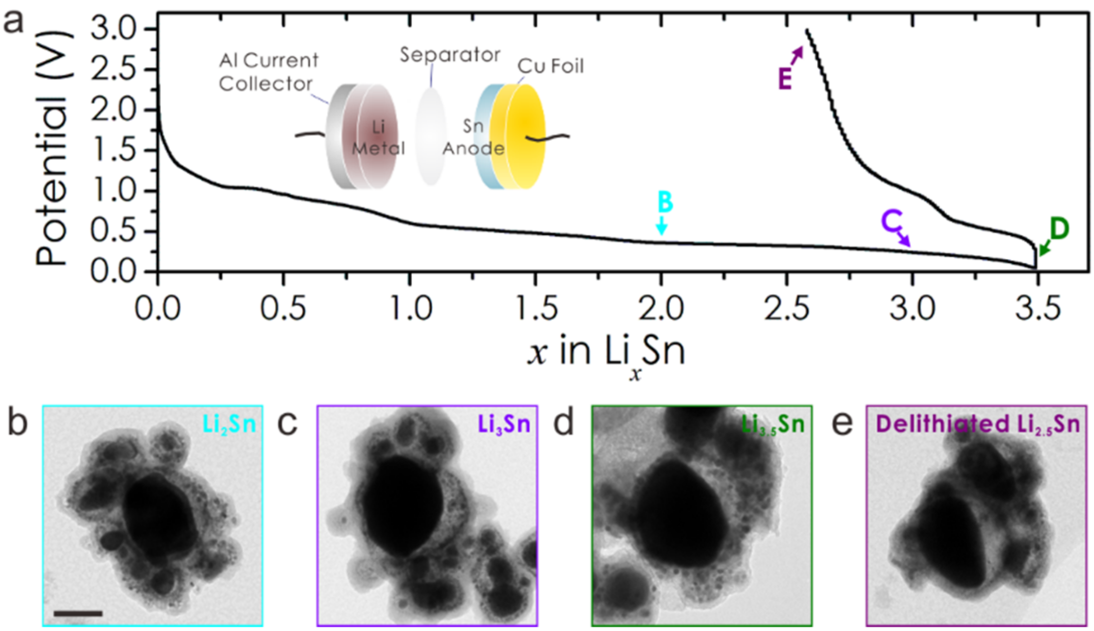

Supplementary Figure 5. *Ex situ* observation of Sn-SnO_2 nanoparticles' morphological evolution during electrochemical cycling against Li. a The charge-discharge curves during the first cycle of Sn-SnO_2 nanoparticles. The inset schematic shows the half-cell configuration. (**b-e**) Representative bright-field TEM images obtained at 4 distinct stages of charge labeled in **a**. The scale bar indicates 200 nm.

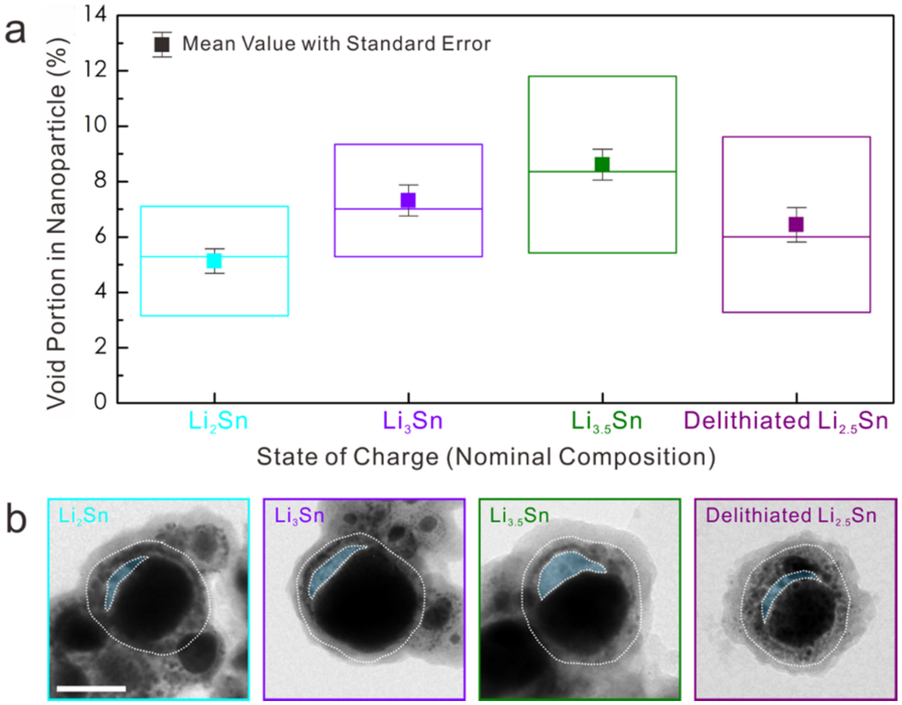

Supplementary Figure 6. Void-to-particle areal ratio according to the state-of-charge. a Void-to-particle areal ratio at the nominal compositions of Li_2Sn (cyan), Li_3Sn (violet), $Li_{3.5}Sn$ (green) and the delithiated $Li_{2.5}Sn$ (purple). The filled squares with error bars indicate the standard deviations. The open boxes show the 25, 50, and 75 percentiles obtained from measurements on 20, 33, 13 and 26 nanoparticles for the corresponding nominal compositions, respectively. **b** BF-TEM images show the void (colored in blue) and total particle (white dashed lines) areas of the representative samples at each composition. The scale bar indicates 200 nm.

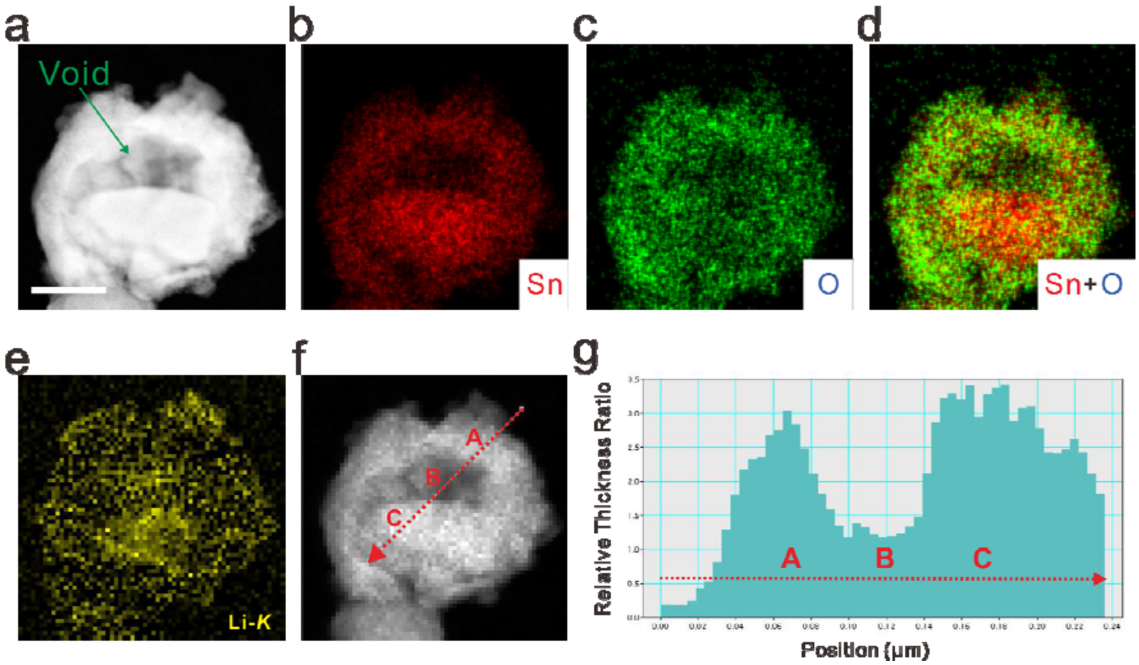

Supplementary Figure 7. Voided morphology of a lithiated Sn-SnO_2 nanoparticle after electrochemical lithiation. a-d HAADF-STEM image and EDS elemental maps (Sn: red, O: blue) show the dealloying-induced voids inside the oxide shell after the 1st lithiation. **e** EELS mapping of Li-K spectra reveal that the void is not Li rich phases formed via phase separation within the particle. **f-g** The thickness profile obtained by EELS spectra across the particle diameter show that the void is indeed a void with significantly reduced material thickness. The scale bar in **a** indicates 100 nm.

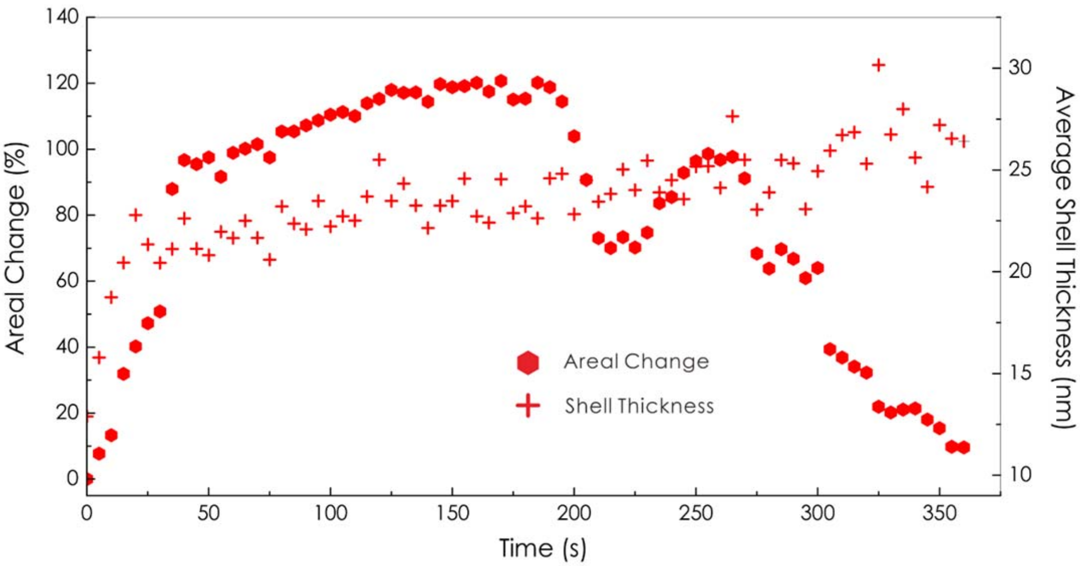

Supplementary Figure 8. The dimensional evolution of a Sn-SnO_2 core-shell nanoparticle with 0.85 core-to-particle ratio (*a*/*b* = 0.85). The changes in the projected core area (red hexagon) with the average oxide shell thickness (red cross) are displayed over the lithiation time.

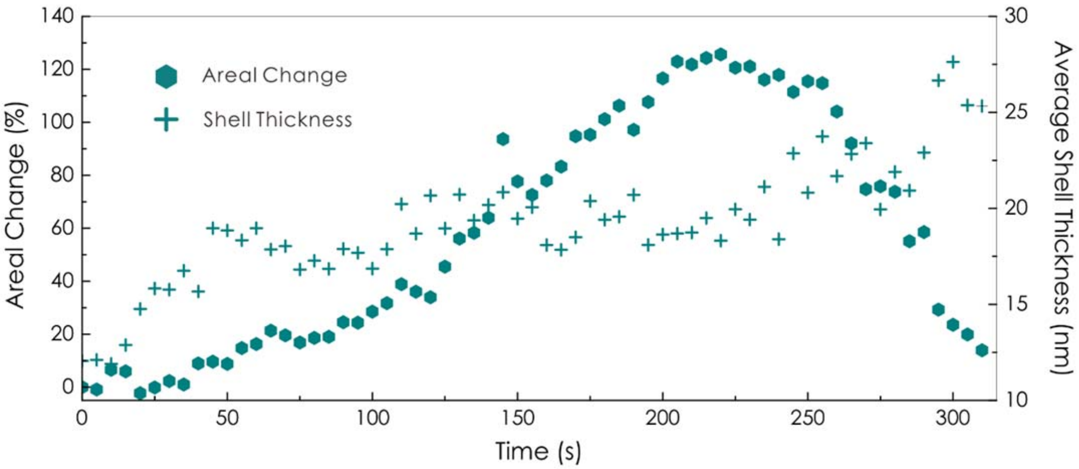

Supplementary Figure 9. The dimensional evolution of a Sn-SnO_2 core-shell nanoparticle with 0.83 core-to-particle ratio (*a*/*b*= 0.83). The changes in the projected core area (dark cyan hexagon) with the average oxide shell thickness (dark cyan cross) are displayed over the lithiation time.

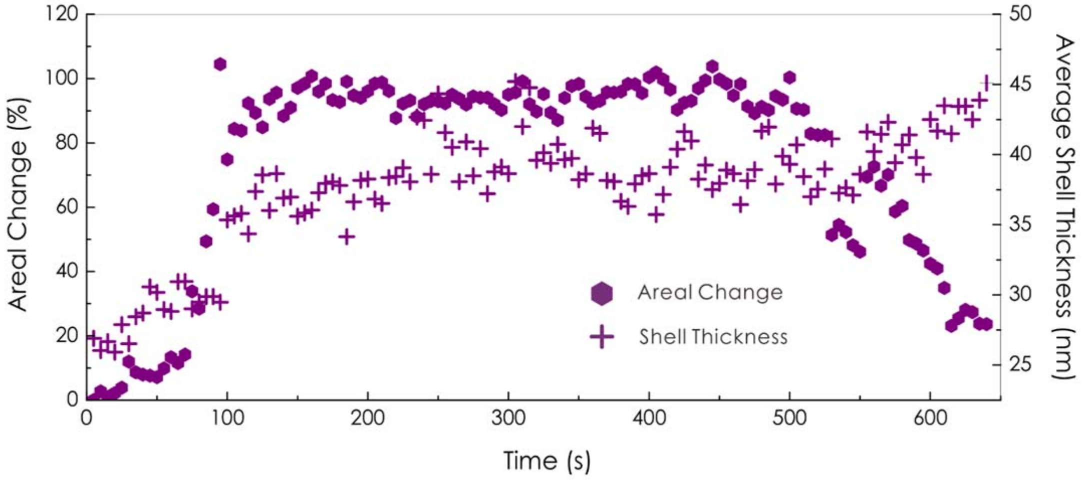

Supplementary Figure 10. The dimensional evolution of a Sn-SnO_2 core-shell nanoparticle with 0.76 core-to-particle ratio (*a*/*b* = 0.76). The changes in the projected core area (purple hexagon symbol) with the average oxide shell thickness (purple cross symbol) are displayed over lithiation time.

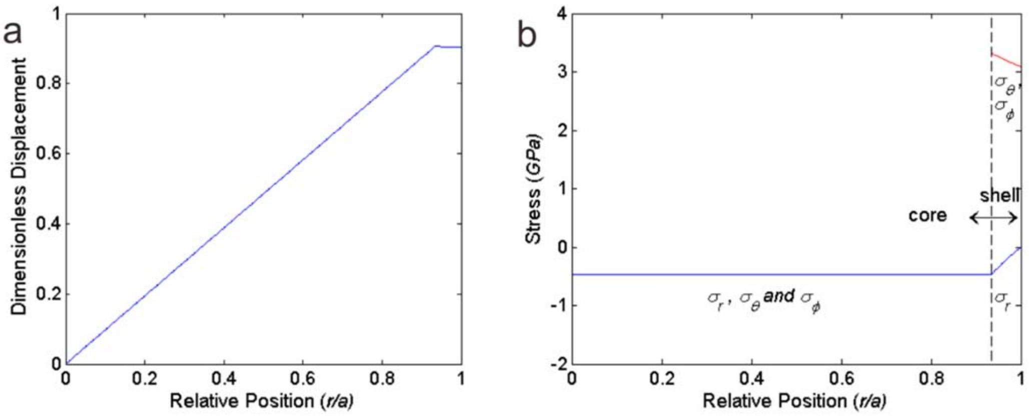

Supplementary Figure 11. The modeled elastic deformation inside the core-shell nanoparticle. a The dimensionless displacement obtained for the relative particle position and **b** the stress components for the core and shell for thin-shelled nanoparticle

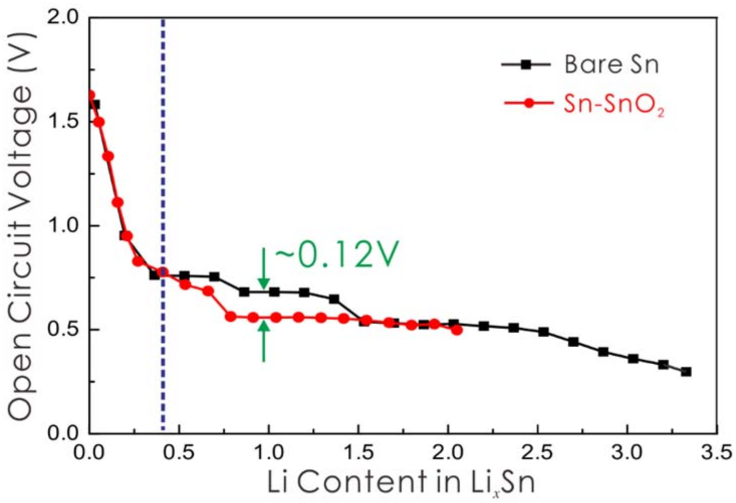

Supplementary Figure 12. The effect of mechanical stress on the equilibrium open circuit voltage (OCV). OCV profiles of bare Sn and Sn-SnO_2 electrodes obtained from GITT measurements are plotted according to the corresponding Li composition (Supplementary Note 8). The OCV values for the two electrodes deviate from each other from around $Li_{0.4}Sn$ composition, reaching up to 0.12 V.

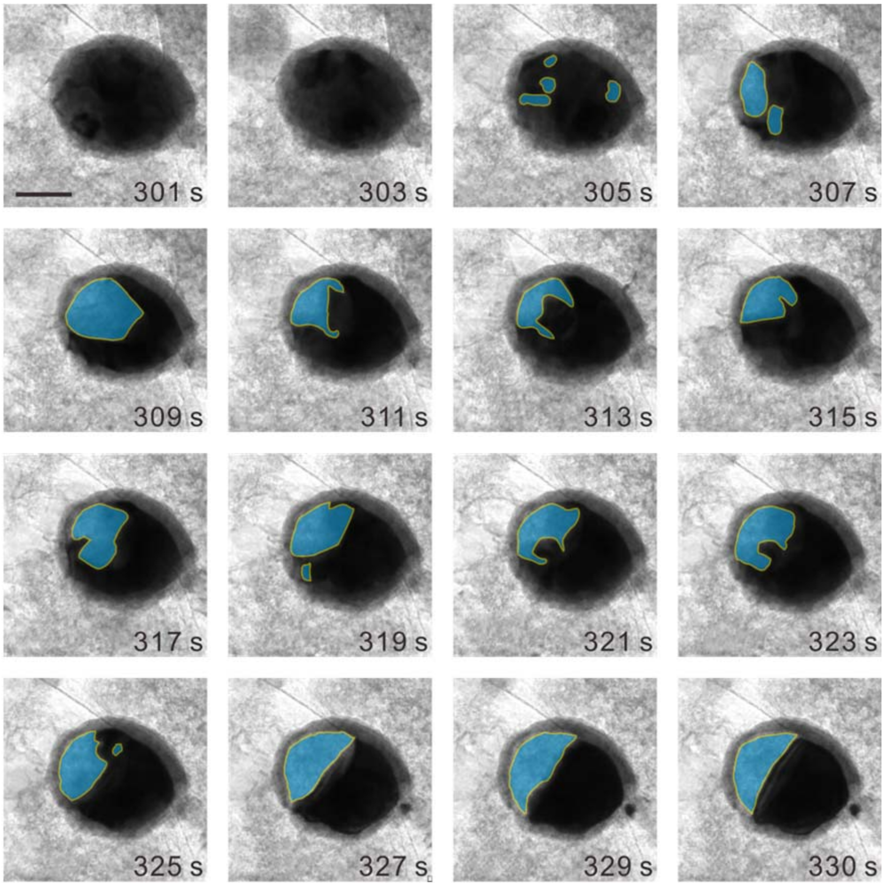

Supplementary Figure 13. Coarsening dynamics of Sn during dealloying. The time-series images of Supplementary Movie 3 (from 301 to 330 s at 2-second intervals) show the morphological evolution during spontaneous dealloying and void formation. Small Kirkendall voids form initially and coarsen to form a stable single meniscus. During this process, dynamic fluctuations in pore volume and curvature are observed. For easy viewing, the pore area is overlaid with blue (outlined by a yellow curve) on each frame. The scale bar indicates 100 nm.

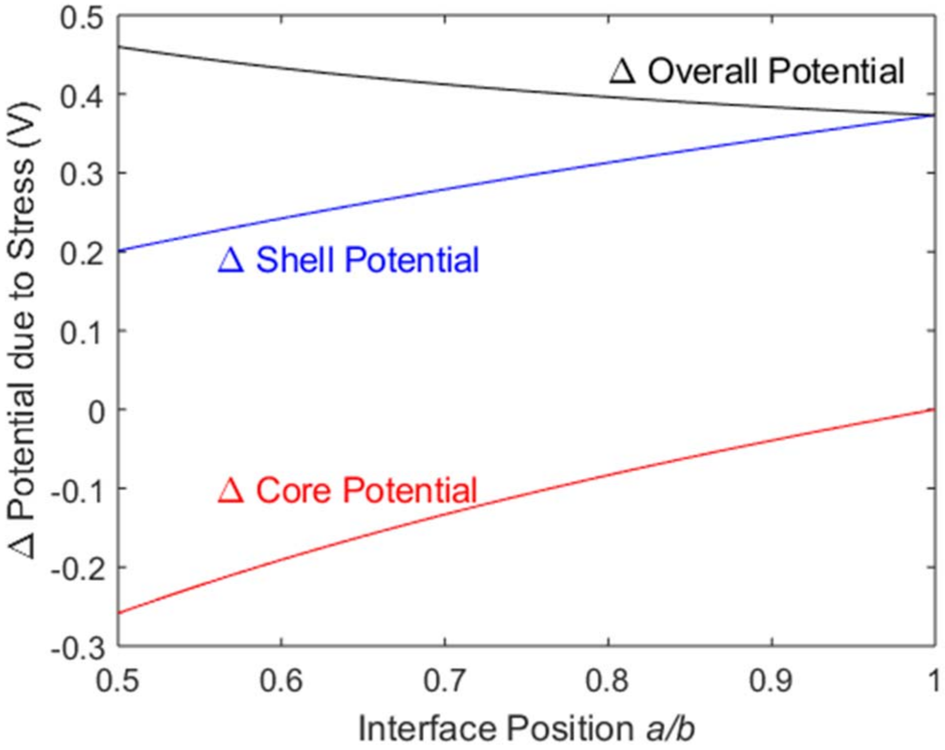

Supplementary Figure 14. Effect of relative shell thickness on the stress contributed potentials. In the thin shell limit, the core does not experience any compression while the shell undergoes tension at the yield stress. As the shell thickens ($a/b = 0.5$), the compression on the core increases. The overall potential difference between the shell and the core reaches 0.5 V at this point.

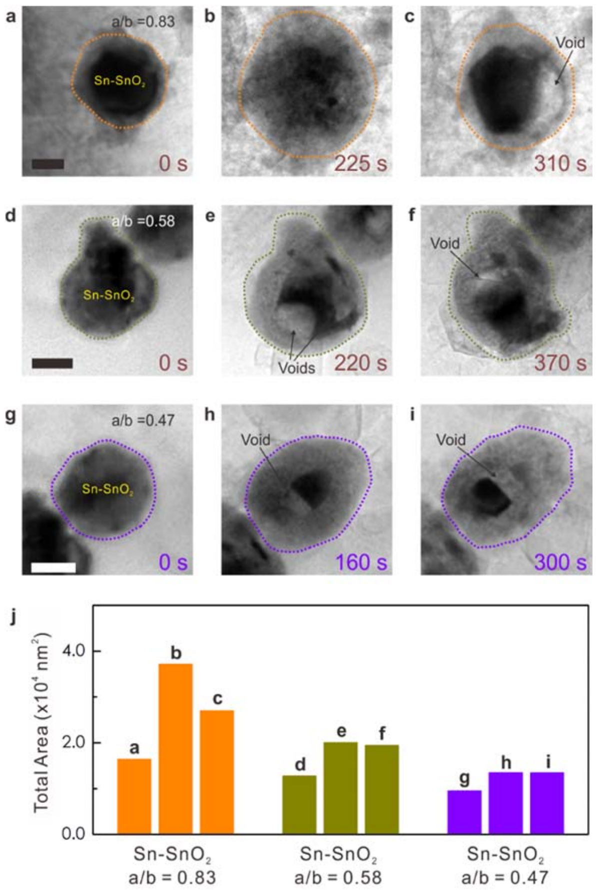

Supplementary Figure 15. The effect of oxide shell thickness on Sn nanoparticles' lithiation morphology. a-c The lithiation of the thin-shelled nanoparticle (*a*/*b*=0.83) forms a pore from core delithiation. **d-f** The lithiation of the thicker-shelled nanoparticle (*a*/*b*= 0.58) forms a pore at an earlier stage of lithiation. **b,e** We observe the thicker-shelled nanoparticle dealloys at 220 s while the thin-shelled nanoparticle show continue lithiation at 225 s. **g-i** The lithiation of the thickest-shelled nanoparticle (*a*/*b*= 0.47) forms a pore at 160 s. Scale bars indicate 50 nm. **j** The particle areas including pore area for each time-series image are plotted for the thin-, thicker-, and thickest-shelled nanoparticles. We observe permanent volume increase even after dealloying.

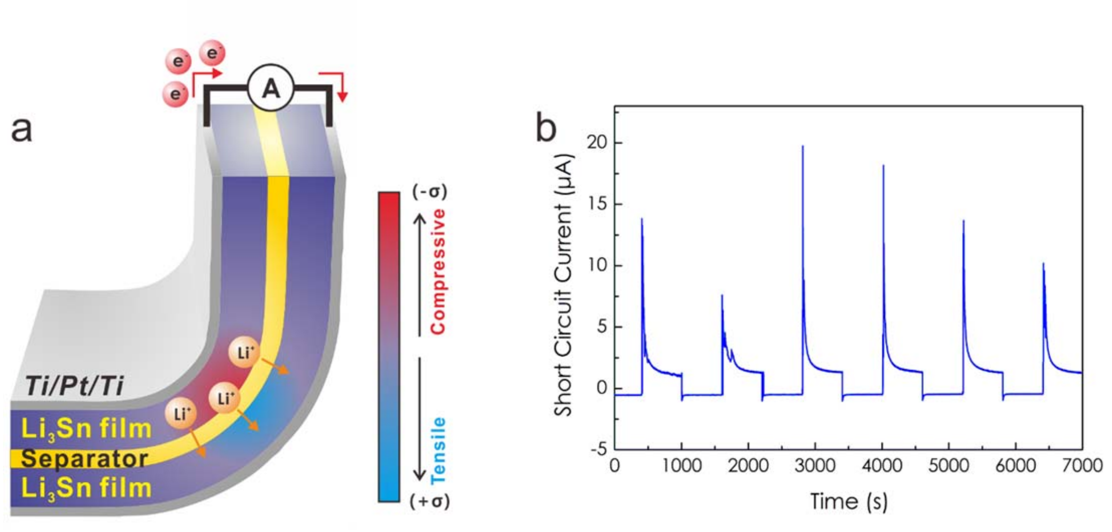

Supplementary Figure 16**. Directional Li migration demonstrated in Li_3Sn thin film-based mechanical energy harvesters**. **a** Schematic showing the stress gradient and the resultant Li migration upon bending the device. **b** The positive short circuit current is generated by periodically applying compression on one Li_3Sn film and tension on the other by bending. The amount of stress applied is approximately 15.6 MPa.

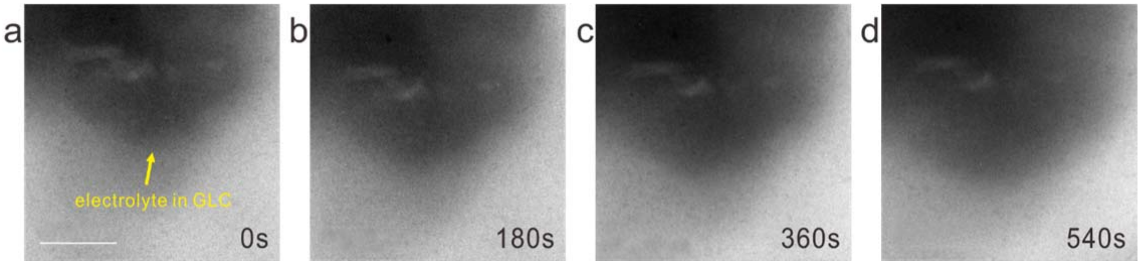

Supplementary Figure 17. The time-series images showing electrolyte bubbles encapsulated by graphene sheets. a-d During continued electron beam irradiation for 540 s, no gaseous bubble or nanoparticle precipitate forms. The scale bar indicates 200 nm.

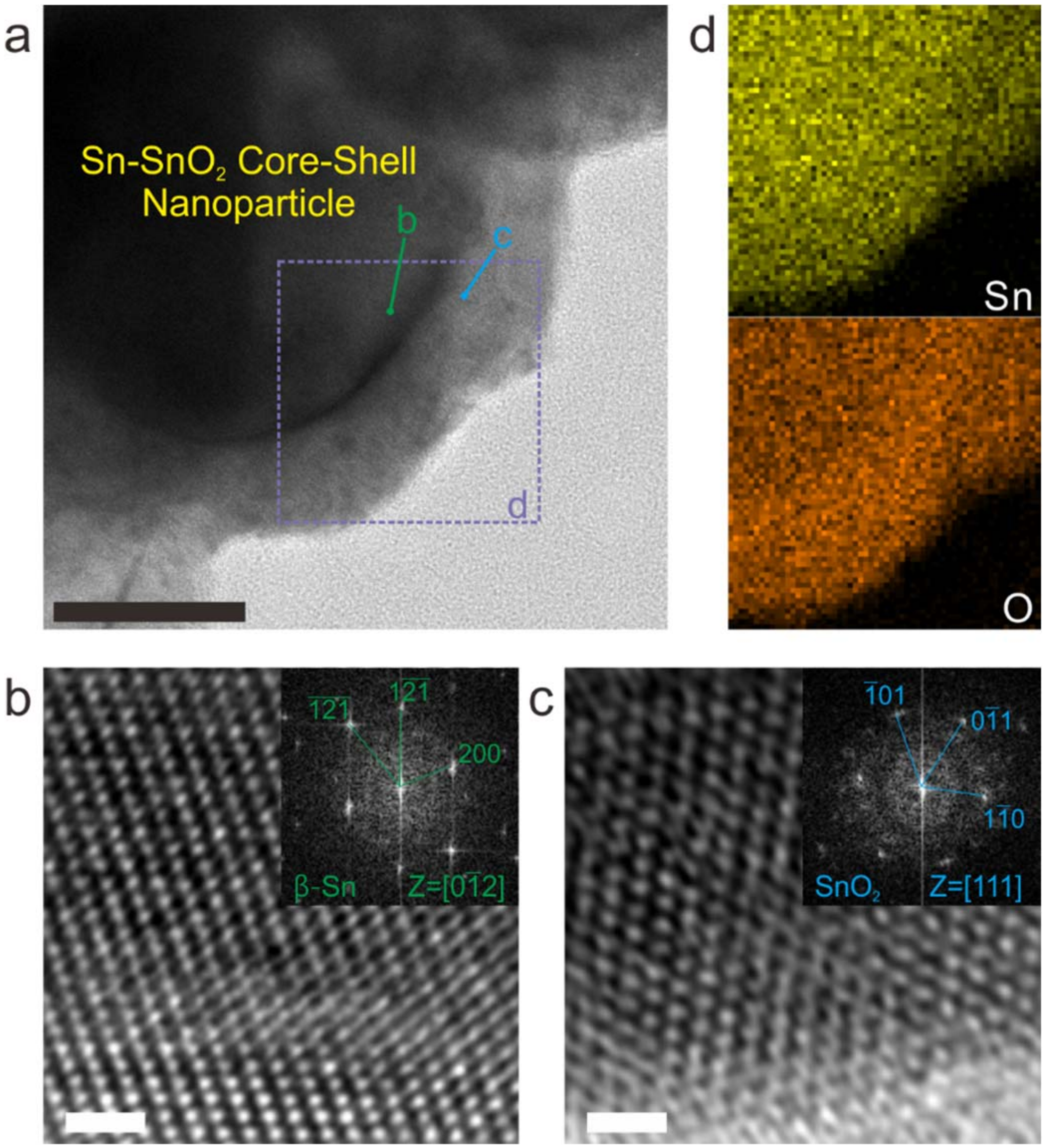

Supplementary Figure 18. Pristine Sn-SnO$_2$ core-shell nanoparticles. **a** A BF-TEM image shows dark contrasted core and bright contrasted oxide shell. The scale bar indicates 50 nm. HR-TEM images on the core **b** and on the shell **c** confirm the crystalline β-Sn and SnO$_2$ in the core and shell, respectively. The scale bars on **b** and **c** indicate 1 nm. **d** EDS analyses show elemental distribution of Sn and O at the purple square region marked in panel **a**.

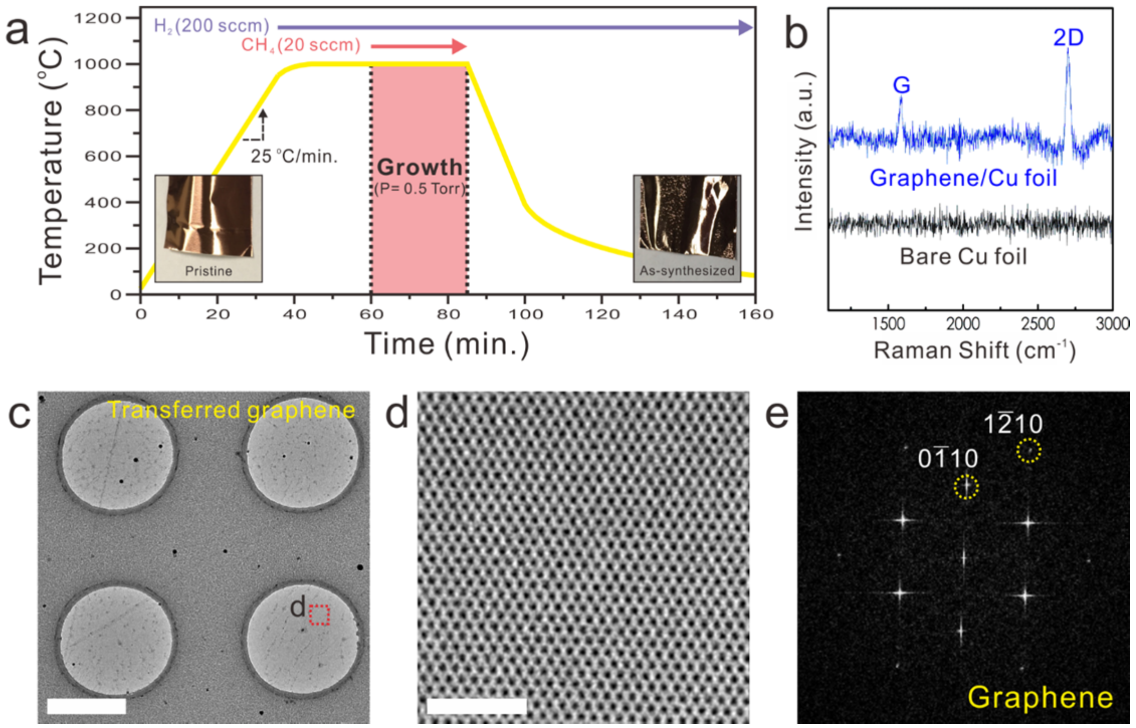

Supplementary Figure 19. Synthesis of CVD-graphene for the liquid cell. a A temperature-time plot for the graphene growth condition by CVD. Inset optical images show the Cu foil before and after graphene growth. **b** Raman spectra of the bare and graphene-covered Cu foil confirm that monolayer graphene is grown. **c** A low-magnification BF-TEM image of the transferred graphene onto a perforated TEM grid coated with carbon membrane. **d**,**e** A high-resolution TEM image and its electron diffraction pattern identifying the synthesized graphene with high crystallinity. Scale bars in **c** and **d** indicate 1 μm and 2 nm, respectively.

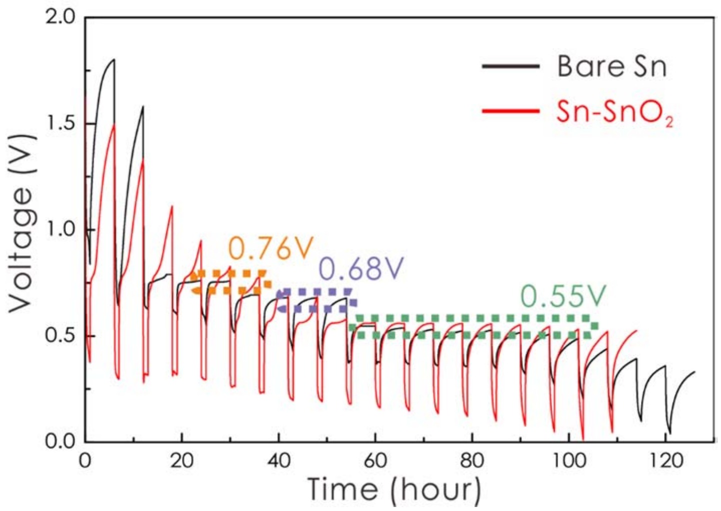

Supplementary Figure 20. The OCV obtained after 5 hour rest periods at different lithiation contents for bare Sn and core-shell Sn-SnO_2 nanoparticles

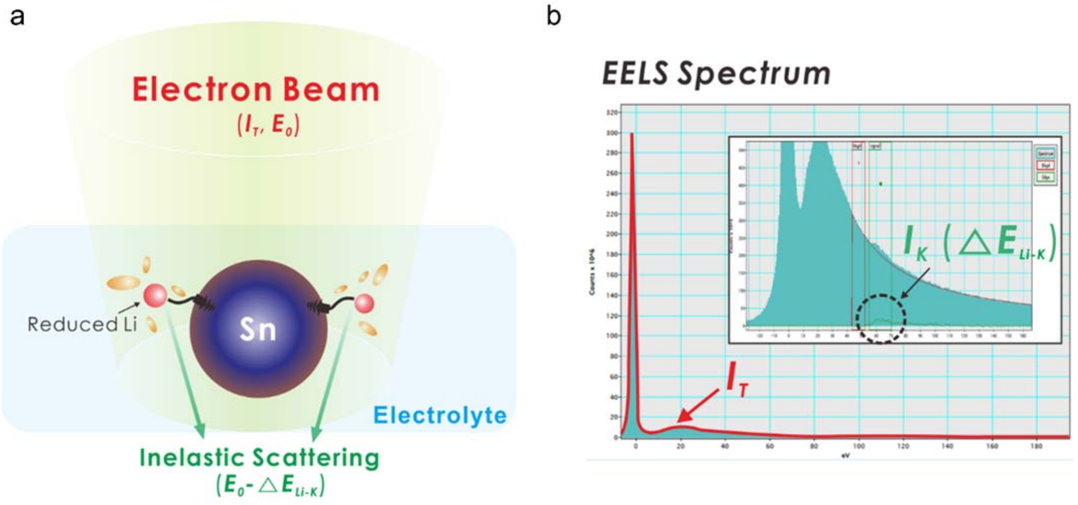

Supplementary Figure 21. Schematics illustrating the mechanism for EELS spectra. a Schematics for electron beam irradiation to the electrolyte bubble and the immersed Sn nanoparticle **b** Examples of the obtained EELS spectra and Li-K edge intensity

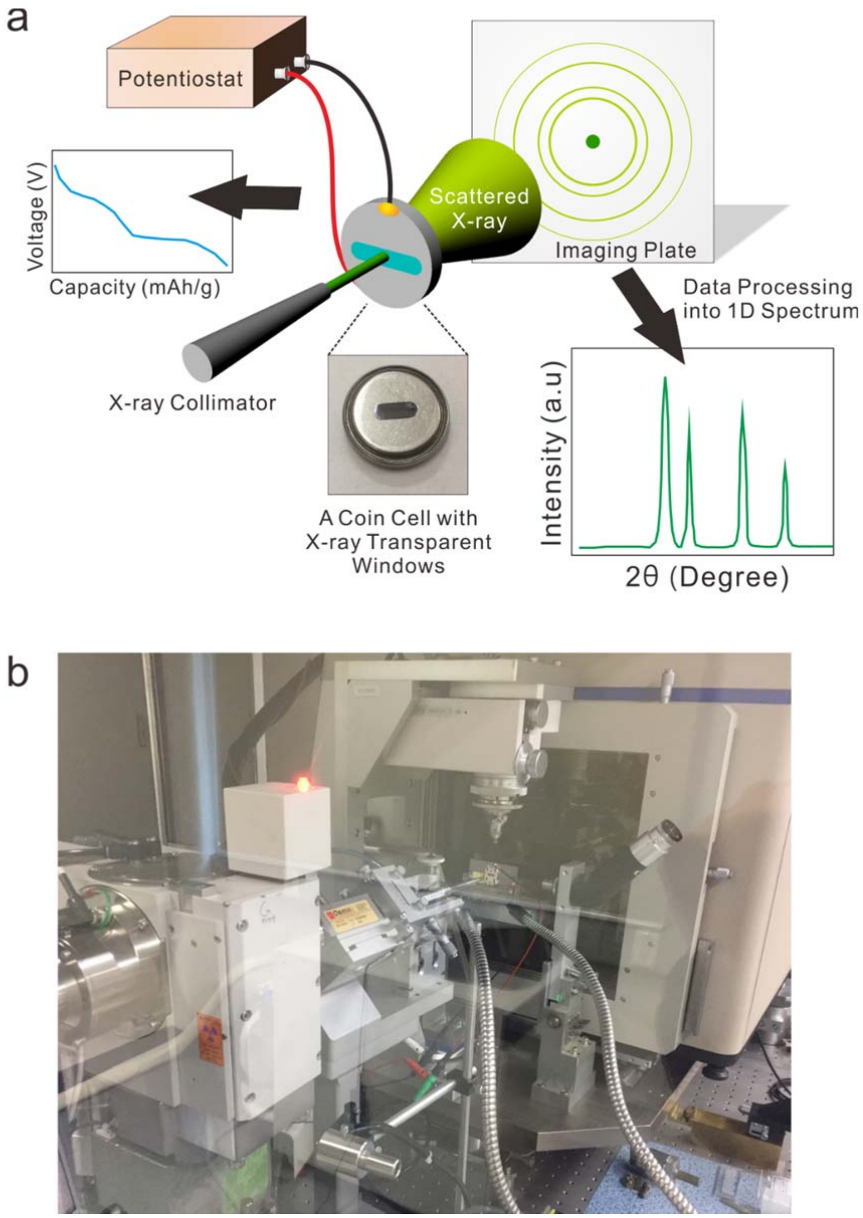

Supplementary Figure 22. Configuration of the *in situ* X-ray scattering experiment. a A schematic illustrating the *in situ* SAXS experiment. **b** Picture of the actual experimental setup.

Supplementary Tables

	Total intensity I_T (Counts, $\times 10^{8}$)	I_K (Counts of Li-K, $\times 10^{6}$)	Absolute thickness (t_{ab}, nm)	Areal density (nm^{-2})	Inelastic scattering cross section (σ_K, $\times 10^{-4}$)
Area 1	5.92	4.26	101.97	61.39	1.17
Area 2	3.96	1.42	67.24	40.28	0.89
Area 3	4.99	2.21	63.89	38.46	1.15

Supplementary Table 1. The obtained intensity and inelastic scattering for three different GLC-EM area

Phase	Space group (No.)	Lattice parameter ($\boldsymbol{a, b, c} / \boldsymbol{\alpha, \beta, \gamma}$)	JCPDS card No.
ß-Sn	$I4_1/amd$ (141)	5.83, 5.83, 3.18<br>90.0, 90.0, 90.0	00-004-0673
α-Sn	$Fd\bar{3}m$ (227)	6.48, 6.48, 6.48<br>90.0, 90.0, 90.0	00-005-0390
SnO_2	$P4_2/mnm$ (136)	4.73, 4.73, 3.18<br>90.0, 90.0, 90.0	00-021-1250
Li_2O	$Fm\bar{3}m$ (225)	4.62, 4.62, 4.62<br>90.0, 90.0, 90.0	00-012-0254
Li_2Sn_5	P4/mbm (127)	10.27, 10.27, 3.12<br>90.0, 90.0, 90.0	01-074-0561
Li_5Sn_2	$R\bar{3}m$ (166)	4.74, 4.74, 19.83<br>90.0, 90.0, 120.0	00-029-0839
Li_7Sn_3	$P2_1/m$ (11)	8.56, 4.72, 9.45<br>90.0, 105.9, 90.0	01-071-9517
$Li_{13}Sn_5$	$P\bar{3}m1$ (164)	4.7, 4.7, 17.12<br>90.0, 90.0, 120.0	00-029-0838
Li_7Sn_2	Cmmm (65)	9.8x13.8x4.75<br>90.0, 90.0, 90.0	00-029-0837
$Li_{22}Sn_5$	F23 (196)	19.78, 19.78, 19.78<br>90.0, 90.0, 90.0	00-018-0753

Supplementary Table 2. Crystal data of pristine Sn, SnO_2 and its lithiated products obtained from Inorganic Crystal Structure Database (ICSD)

	Core volume ($\times 10^6$ nm^3)	Core mass (M, $\times 10^{-14}$ g)	Estimated C-rate	Electron beam density ($\times 10^{-14}$ A nm^{-2})
core-shell (a/b=0.85)	2.53	1.84	17.0	7.71
core-shell (a/b=0.76)	2.47	1.79	6.40	3.01
core-shell (a/b=0.83)	0.91	0.66	19.4	7.71

Supplementary Table 3. The comparison between the estimated C-rates and electron beam density

Parameter	Notes
$\bar{\boldsymbol{\theta}}_{\boldsymbol{\alpha}} = \mathbf{0}$	Diffusion-limited behavior
$\boldsymbol{\xi} = \mathbf{1}$	Similar Li diffusion coefficient between core and shell
$\boldsymbol{K} = \mathbf{1}$	Similar core and shell activity coefficient
$\bar{\boldsymbol{\Omega}} = \mathbf{0.5}$	Modest volume expansion in shell upon solute addition
$\bar{\boldsymbol{\Gamma}}_{\boldsymbol{\alpha\beta}} = \mathbf{0}$	Small interfacial energy between core and shell
$\bar{\boldsymbol{\Gamma}}_{\boldsymbol{\beta}} = \mathbf{0}$	Large particle and small surface energy
$\bar{\boldsymbol{E}} = \mathbf{1.1}$	Elastic modulus ratio of shell (36.3 GPa) to core (33.3 GPa)
$\boldsymbol{\nu}_{\boldsymbol{\alpha}} = \mathbf{0.36}$	Poisson ratio of the core
$\boldsymbol{\nu}_{\boldsymbol{\beta}} = \mathbf{0.36}$	Poisson ratio of the shell
$\boldsymbol{\chi}_{\mathbf{i}} = \mathbf{0.93}$	Core to shell thickness ratio
$\boldsymbol{x}_{\mathbf{T},\boldsymbol{\alpha}} = \mathbf{0.4}$	Composition *x* in Li_xSn

Supplementary Table 4. The major variables and their values employed in the elasticity models

Supplementary Note

1. Graphene liquid cell for studying Li alloying dynamics in Sn nanoparticles

Supplementary Fig. 1a and 1b schematically describe the GLC-EM for investigating lithiation-induced morphological and phase evolutions of Sn-SnO_2 core-shell nanoparticles immersed in a commercial electrolyte solution. Graphene sheets served as robust membranes separating the specimen in volatile liquid from a high vacuum environment inside the TEM (the pressure range of ~10^{-6}-10^{-9} Torr). Simultaneously, the thinnest graphene viewing window mitigates the undesirable deterioration in TEM image contrasts through the wet specimen[1]. Energetic electron beams irradiated for imaging provides electrical current input to drive the lithiation of Sn nanoparticles[2], similar to the galvanostatic lithiation condition. A scanning transmission electron microscopy (STEM) image of the fabricated liquid cell is shown in Supplementary Fig. 1c. Sn nanoparticles in the commercial electrolyte bubble are lithiated with continuous electron beam irradiation for 356 seconds. The volume expansion and agglomeration processes are clearly observed in Supplementary Fig. 1d.

2. Lithiation condition inside graphene liquid cell during *in situ* TEM observation

Tracking the lithiation dynamics inside GLC reveals that the particle expands linearly over time from the beginning of lithiation (Supplementary Fig. 1, 4, 8-10). When two similar particles are lithiated at identical lithiation rates, we observe similar lithiation dynamics (Supplementary Fig. 4). The slope changes once the particles get heavily lithiated, likely due to the increased volume of the particle. These results suggest that the lithiation inside GLC occurs with constant Li injection, similar to the galvanostatic lithiation condition.

From electron energy loss spectroscopy (EELS) theory, the inelastic scattering cross section of Li K-edge (σ_K) is defined as follow,

$$\sigma_{\mathrm{K}} = \frac{I_{\mathrm{K}}}{N I_{\mathrm{T}}} \quad (1)$$

I_{T} indicates the total incident current intensity, which is sum of the total energy loss in an EELS spectrum (Supplementary Fig. 20). I_{K} and N respectively indicate the background-subtracted Li K-edge signal intensity and the areal Li density. To measure the absolute liquid electrolyte thickness (t_{ab}) from the EELS spectrum, we directly employ the log-ratio formula algorithm in Gatan Digital Micrograph with the experimental parameters such as the accelerating voltage (300 kV), the semi-convergence and the semi-collection angles (12 and 20 mrad, respectively), and the effective atomic number (5.12, for carbonates solvents)[6]. For 1M $LiPF_6$ electrolyte, we assume uniform Li distribution in the encapsulated electrolyte bubble (0.602 Li in 1 nm^3 electrolyte volume) and the areal density is approximated as the concentration of the electrolyte multiplied by the measured t_{ab}.

We then obtain the I_{T}, I_{K}, and t_{ab} from three selected areas and calculate the inelastic scattering cross section (σ_{K}), as shown in the Supplementary table 2.

It is noted that the reactive Li species activated by the inelastic interaction are approximately 0.01% in the electrolyte and they predominantly initiate the galvanostatic lithiation in GLC since the insulating oxide shells are expected to limit the electron transport from the irradiated Sn particles required for direct lithiation (Supplementary Fig. 1). The relatively constant σ_{K} also implies that we can effectively control the lithiation rate (C-rate) by adjusting the electron beam current density irradiated on the GLC.

3. Voided morphologies in electrochemically lithiated Sn-SnO_2 nanoparticles

The voided morphologies are observed at all four stages of first cycling, with the nominal compositions of lithiated Li_2Sn, Li_3Sn, $Li_{3.5}Sn$ and delithiated $Li_{2.5}Sn$ (Supplementary Fig. 5). The average portion of voids increases from 4.3% to 9.7% as the

nominal composition increases from Li_2Sn to $Li_{3.5}Sn$ (Supplementary Fig. 6), which suggests that the void size is affected by Li concentration in lithiated Sn-SnO_2 nanoparticles. The voided morphology is consistently observed among different-sized particles with radii ranging from 50 nm to 150 nm. For the fully lithiated particle, we confirm the void formed inside the lithiated shell (Supplementary Fig. 7). After the galvanostatic delithiation, the particles exhibit similar core morphology, but with noticeably thinner shells than those in lithiated particles (Supplementary Fig. 5e).

4. Area measurements and C-rate estimations

The area of the core-shell nanoparticles was measured by counting the pixel number of the selected contrast contour using ImageJ (National Institutes of Health). The error bound for measuring lithiation-induced areal changes in nanoparticles were ±3.4% (Fig. 2).

The electron beam-induced C-rate during our *in situ* GLC tests were calculated based on the initial volume increase rate. Since the volume change during lithiation of Sn alloy is linearly proportional to the Li composition[7,8], the C-rate can be estimated from volume change rate during lithiation. To ensure that the lithiation rate is controlled, electron beam irradiation dose is maintained constant. We use the first observed maximum in core area as a reference point and measure the areal change (ΔA) and lithiation time. The volume change is then estimated assuming that the nanoparticle remains spherical. For the nanoparticle in Fig. 2, the measured core areas of pristine and lithiated states (at 185 s) correspond to 28,893 and 63,618 nm^2, respectively. The volume change (ΔV) is computed as follows:

$$\text{Volume change } (\Delta V, \%) = \frac{V_{\text{lithiated}} - V_{\text{pristine}}}{V_{\text{pristine}}} \times 100$$

$$= \frac{{r_{\text{lithiated}}}^3 - {r_{\text{pristine}}}^3}{{r_{\text{pristine}}}^3} \times 100$$

$$= \frac{(A_{\mathrm{lithiated}})^{1.5} - (A_{\mathrm{pristine}})^{1.5}}{(A_{\mathrm{pristine}})^{1.5}} \times 100$$

$$= \frac{(63{,}618)^{1.5} - (28{,}893)^{1.5}}{(28{,}893)^{1.5}} \times 100 = 227\ \% \qquad (2)$$

V, r, and A indicate the core volume, radius, and area, respectively. The nanoparticle in Fig. 2 is lithiated up to 227 % volume expansion for 185 s. By linear extrapolation, it is inferred that full lithiation takes 212 s under identical electron beam irradiation.

$$\frac{260\ \%\ \text{volume increase}}{227\ \%\ \text{volume increase}} \times 185\ \text{s} = 212\ \text{s}$$

The estimated C-rate is thus 17 C.

Using the same method, we estimate the lithiation rate for the other two core-shell nanoparticles in Fig. 2k to be 6.4 and 19.4 C under 3.01×10^{-14} and 7.71×10^{-14} A nm^{-2} electron beam density, respectively.

The Supplementary Table 3 compares the estimated C-rate to the irradiated electron beam density. As the electron beam density decreases from 7.71×10^{-14} A nm^{-2} to 3.01×10^{-14} A nm^{-2}, the estimated C-rate decreases accordingly. When the electron irradiation is maintained the same, we still observe different lithiation rates for different-sized particles. For smaller particles, the C-rate increases as the Li intake current is identical. Following our analysis on the constant inelastic scattering cross section, this result suggests that we can control the lithiation rate by the irradiated electron beam density for given nanomaterials.

5. Lithiation dynamics of Sn nanoparticles

The lithiation dynamics observed in the *in situ* GLC-EM experiments reveal distinct kinetic regions in lithiation dynamics. The lithiation dynamics plotted in Fig. 2k exhibits rapid initial increase in particle size, followed by a slow yet steady increase before core dealloying begins (pointed by dashed line for each curve). The observed step-wise increase in particle size is similar to those reported for Sn nanowires during potentiostatic lithiation[9]. It is also noted that this distinct two-stage kinetics is observed for both oxide-shelled and pristine Sn particles suggesting that compressive stress does not significantly affect the lithiation kinetics (Fig. 2k and Supplementary Fig. 4b).

It means that the initial lithiation behavior is controlled by lithiation of Sn core. The consistent lithiation kinetics, observed in Sn particles with or without oxide shell, might be due to rapid Li insertion into Sn core through defective grain boundaries[10]. This continuous two-step lithiation dynamics, observed in both oxide-shelled and pristine Sn particles, differs from the initial two phase dynamics in Si.

At the areal expansion up to 100% for the three nanoparticles in Fig. 2k, the rigid oxide shell is expected to break apart. Instead, the shell thickens continuously and demonstrates that Li intake occurs in the shell as well as in the core. As the shell remains intact, the expanding core presumably imposes tensile hoop stress on the shell while the shell imposes compressive hydrostatic stress on the core.

6. Elasticity models for core-shell particles

To identify when the elastic limit is reached for the SnO_2 shell during lithiation, we employed the diffusion-induced elasticity model by Verbrugge *et al.*[11]. Understanding the elastic stress of core-shell geometry during lithiation involves solving the following set of dimensionless differential equations in terms of the displacement ($\bar{u}$). The particle geometry is assumed to be spherically symmetric.

$$\frac{d^2\bar{u}_\alpha}{d\bar{r}^2} + \frac{2}{r}\frac{d\bar{u}_\alpha}{d\bar{r}} - \frac{2\bar{u}_\alpha}{\bar{r}^2} = 0 \quad \text{for } 0 < \bar{r} < \bar{r}_\mathrm{i} \tag{3}$$

$$\frac{d^2\bar{u}_\beta}{d\bar{r}^2} + \frac{2}{\bar{r}}\frac{d\bar{u}_\beta}{d\bar{r}} - \frac{2\bar{u}_\beta}{\bar{r}^2} = 0 \quad \text{for } \bar{r}_\mathrm{i} < \bar{r} < 1 \tag{4}$$

The dimensionless displacement is defined as the following:

$$\bar{u} = \frac{u}{\frac{b\Omega_\alpha x_{\mathrm{T},\alpha}}{3}} \tag{5}$$

Here, α refers to the core phase and β the shell phase. $\bar{r}$ and $\bar{r}_\mathrm{i}$ refer to the dimensionless radii and interface position. b, Ω_α and $x_{\mathrm{T},\alpha}$ refer to the particle radius, partial molar volume of Li in the core and the relative composition x in Li_xSn, respectively. Verbrugge *et al.* shows the analytical solution to the system, with a set of material parameters[29]. The Supplementary Table 4 organizes the constants used in the model. The definitions of these materials properties can be found in the original paper[11]. The physical properties of Li-Sn alloy compounds such as Young's modulus are obtained with Vegard's law (Supplementary Table 4).

The core to shell thickness ratio is obtained from 30 s image for Supplementary Movie 3. The interface between the core and the shell is expected to be non-coherent, since the shell undergoes complete conversion reaction from crystalline SnO_2 into lithiated Sn islands in amorphous Li_2O matrix. This conversion reaction involving nucleation and growth is expected to eliminate the possible coherency strain between the Sn core and the SnO_2 shell. Thus, the interfacial energy is assumed very small. Including the positive surface energy of the shell (β) phase into the model increases the compressive stress exerted on the core, while maintaining the interfacial stress jump across the core-shell boundary. We excluded this to ensure that we do not overestimate the compressive stress on the core. Solving the

displacement equations results in the displacement profile during lithiation (Supplementary Fig. 11a).

While the shell thickness remains approximately unchanged, as the Poisson contraction is countered by the volume expansion induced by lithiation. Noting that the displacement in spherically symmetrical particle is related to the following equation,

$$\epsilon_r = \frac{du}{dr} \quad \text{and} \quad \epsilon_\theta = \frac{u}{r} \tag{6}$$

we may express the dimensionless stresses as the following:

$$\bar{\sigma}_{r,\alpha} = (1-\nu_\alpha)\frac{d\bar{u}_\alpha}{d\bar{r}} + 2\nu_\alpha\frac{\bar{u}_\alpha}{\bar{r}} - (1+\nu_\alpha)\bar{\Theta}_\alpha \tag{7}$$

$$\bar{\sigma}_{\theta=\phi,\alpha} = \frac{\bar{u}_\alpha}{\bar{r}} + \nu_\alpha\frac{d\bar{u}_\alpha}{d\bar{r}} - (1+\nu_\alpha)\bar{\Theta}_\alpha \tag{8}$$

$$\bar{\sigma}_{r,\beta} = \bar{E}\left\{(1-\nu_\beta)\frac{d\bar{u}_\beta}{d\bar{r}} + 2\nu_\beta\frac{\bar{u}_\beta}{\bar{r}} - (1+\nu_\beta)\bar{\Omega}\bar{\Theta}_\beta\right\} \tag{9}$$

$$\bar{\sigma}_{\theta=\phi,\beta} = \bar{E}\left\{\frac{\bar{u}_\beta}{\bar{r}} + \nu_\beta\frac{d\bar{u}_\beta}{d\bar{r}} - (1+\nu_\beta)\bar{\Omega}\bar{\Theta}_\beta\right\} \tag{10}$$

The dimensionless stress components may be converted back to the stress components as the following:

$$\sigma = \frac{\Omega_\alpha x_{\mathrm{T},\alpha} E_\alpha}{3(1+\nu_\alpha)(1-2\nu_\alpha)}\bar{\sigma} \tag{11}$$

We thus obtain the elastic stresses in the particle at $Li_{0.4}Sn$ composition as shown in Supplementary Fig. 11b.

At the composition of $Li_{0.4}Sn$, the core experiences the hydrostatic stress of -0.47 GPa while the shell undergoes tangential stresses just exceeding 3 GPa. Assuming the yield stress of 3 GPa for the SnO_2 shell, we observe that the shell begins to deform plastically at the core composition of $Li_{0.4}Sn$. This clearly shows the need for plasticity models for further-lithiated core-shell particles.

7. Plasticity models for core-shell particles

The lithiation-induced plastic deformation in core-shell particles has been modeled by Zhao *et al.*[12]. The mechanical equilibrium condition for radial (σ_r) and tangential ($\sigma_\theta = \sigma_\phi$) components in spherically symmetric system runs as follows.

$$\frac{\partial \sigma_r(r,t)}{\partial r} + 2\frac{\sigma_r(r,t) - \sigma_\theta(r,t)}{r} = 0 \quad (12)$$

As the shell deforms plastically, the stress states follow the state equation $\sigma_\theta - \sigma_r = \sigma_{\mathrm{Y}}$, where σ_{Y} indicates the yield stress. Thus, the mechanical equilibrium reduces to the following.

$$\frac{\partial \sigma_r(r,t)}{\partial r} - 2\frac{\sigma_{\mathrm{Y}}}{r} = 0 \quad (13)$$

Applying the boundary conditions of the traction-free surface, we obtain the following solutions for the shell:

$$\sigma_r = -2\sigma_{\mathrm{Y}} \log\left(\frac{b}{r}\right), \text{for } a \le r \le b \quad (14)$$

$$\sigma_\theta = \sigma_{\mathrm{Y}} - 2\sigma_{\mathrm{Y}} \log\left(\frac{b}{r}\right), \text{for } a < r \le b \quad (15)$$

Here, a and b refer to the core and particle radii, respectively. Similarly, in the core, we obtain the following expressions for the stress states:

$$\sigma_r = \sigma_\theta = \sigma_\phi = -2\sigma_{\mathrm{Y}} \log\left(\frac{b}{a}\right), \text{for } r \le a \quad (16)$$

The solutions to the plasticity model only contains one materials parameter, the yield stress of the shell. Solving the equations for the particle's materials parameters gives the stress states in Fig. 4a.

8. GITT measurements of bare Sn and Sn-SnO_2 electrodes

To measure the stress contribution to equilibrium OCV, GITT measurements were carried out on pristine Sn and core-shell Sn-SnO_2 electrodes by periodical applying 1-hour-long galvanostatic current (0.05 C) between 5-hour-long rest time. Orange, purple, and green dotted lines note the curve sections with the OCV values of 0.76, 0.68 V and 0.55 V, respectively. Based on the GITT measurements, we plotted the OCV curves with Li composition for Sn and Sn-SnO_2 nanoparticles (Supplementary Fig. 12). We observe that approximately 0.12 V difference arises between the two particle types from $Li_{0.4}Sn$ composition.

9. The amount of Li migration from core to shell

For the particle with a/b ratio of 0.85, we examine whether the shell may accommodate all the Li from the core's composition change. For the spherical particle geometry, the particle radii and shell thickness are 84 nm and 15 nm, respectively. Assuming the particle is fully dense, the core has 1.5×10^{-16} moles of Sn and the shell 7.3×10^{-16} moles of SnO_2. The composition in the core changes from $Li_{3.5}Sn$ to $Li_{0.4}Sn$, equivalent to approximately 4.7×10^{-16} moles of Li. The limiting amount of Li the shell can take according to the balanced equation ($SnO_2 + 8.4Li \rightarrow 4Li_2O + Li_{4.4}Sn$) is 5.2×10^{-16} moles of Li, ensuring that all Li ejected from the core can be accommodated in the shell.

10. Effect of shell to core ratio on stress contributed potential

Supplementary Fig. 14 plots the potential contributions due to the shell-imposed stresses with respect to the shell thickness. At the thin shell limit, the shell-imposed stress on the core (and the equivalent stress potential) converges to zero. The shell experiences stress potential corresponding to the yield stress.

Increasing the shell thickness results in enlarged potential drop on the core. In the thin shell case, the potential drop on the core is negligible, while the potential increase on the shell remains significant. The difference between the two potential curves, labeled as the overall potential difference, increases with increasing shell thickness.

11. Coarsening dynamics in the dealloyed Sn

In the *in situ* nanoparticle experiments, since the diffusivity of Li is much higher at room temperature than that of Sn, the interdiffusivity difference causes Li to migrate faster, resulting in Kirkendall voiding. Here, we analyze the exciting temporal dynamics of this void evolution from vacancies and show that such dynamic void evolution in real time is visible only when the particle size is below 400 nm.

Gibbs-Thompson equation describes the chemical potential of a material according to the radius of curvature (r),

$$\mu_r = \mu_\infty + \gamma\kappa v_{\mathrm{m}} = \mu_\infty + \gamma v_{\mathrm{m}}\left(\frac{1}{r_1} + \frac{1}{r_2}\right) \quad (17)$$

where μ_∞ indicates the chemical potential for bulk materials, γ surface energy, κ the curvature and v_{m} atomic volume. For spherical particles with radius r, the chemical potential (μ_{s}) increases compared to the bulk chemical potential.

$$\mu_{\mathrm{s}} = \mu_\infty + \gamma v_{\mathrm{m}}\left(\frac{2}{r}\right) > \mu_\infty \quad (18)$$

For porous materials, the negative curvature results in reduced chemical potential (μ_{p}) compared to the bulk.

$$\mu_{\mathrm{p}} = \mu_\infty - \gamma v_{\mathrm{m}}\left(\frac{2}{r}\right) < \mu_\infty \quad (19)$$

A spherical particle with voids, therefore, results in material flow from the particle surface to the voids, filling in the voids. Assuming the voids are interconnected, we may derive the following expression for the surface diffusion flux (J):

$$J = \frac{c_{\mathrm{surface}} D_{\mathrm{surface}}}{kT}\left(\frac{\partial \mu}{\partial x}\right) = \frac{\gamma v_{\mathrm{m}} D_{\mathrm{surface}}}{kT}\left(\frac{\partial^3 h}{\partial x^3}\right) \qquad (20)$$

Here, c_{surface} and D_{surface} indicate the surface concentration and the diffusion coefficient of the diffusing species, respectively. k, T and h refer to Boltzmann constant, temperature and the height function describing the surface morphology, respectively. Solving the equations above to obtain the coarsening dynamics, we obtain the following relation:

$$\frac{\partial h}{\partial t} = -\nabla \cdot (\Omega J \delta) = -\left(\frac{\gamma \delta \Omega D_{\mathrm{surface}}}{kT}\right)\frac{\partial^4 h}{\partial x^4} \qquad (21)$$

$$h(t) = h(0)e^{-B\left(\frac{2\pi}{\lambda}\right)^4 t} \qquad (22)$$

Here, Ω and δ indicate the molar volume of the diffusing species and the surface layer thickness, respectively. λ refers to the characteristic feature size, here the void size. B is the grouped constants. As the feature size doubles, we note that coarsening time increases by 16-fold. This indicates that controlling the particle sizes acts as a key to preventing pulverization. We observe sintering 10 nm-scale Kirkendall voids takes approximately 25 seconds (Supplementary Fig. 13). Sintering 400 nm pores would take 740 days to sinter, explaining the size-dependent nanostructure formation in electrochemically dealloyed Li_xSn[13]. Cycling large Sn particles involves both increased stresses and pores too large to be sintered *via* surface diffusion; the pores likely act as crack nucleation sites *via* stress concentration. This size-dependence suggests certain recovery mechanisms activate only at nanoscales[14,15] and designing Sn's feature sizes may significantly affect the particle's mechanical integrity[16].

Supplementary References

1 Yuk, J. M. *et al.* High-resolution EM of colloidal nanocrystal growth using graphene liquid cells. *Science* **336**, 61-64 (2012).

2 Yuk, J. M., Seo, H. K., Choi, J. W. & Lee, J. Y. Anisotropic lithiation onset in silicon nanoparticle anode revealed by *in situ* graphene liquid cell electron microscopy. *ACS Nano* **8**, 7478-7485 (2014).

3 Kresse, G. & Furthmüller, J. Efficient iterative schemes for ab initio total-energy calculations using a plane-wave basis set. *Phys. Rev. B* **54**, 11169-11186 (1996).

4 Perdew, J. P., Burke, K. & Ernzerhof, M. Generalized gradient approximation made simple. *Phys. Rev. Lett.* **77**, 3865-3868 (1996).

5 Blöchl, P. E. Projector augmented-wave method. *Phys. Rev. B* **50**, 17953-17979 (1994).

6 Leenheer, A. J., Jungjohann, K. L., Zavadil, K. R., Sullivan, J. P. & Harris, C. T. Lithium electrodeposition dynamics in aprotic electrolyte observed *in situ via* transmission electron microscopy. *ACS Nano* **9**, 4379-4389 (2015).

7 Obrovac, M. N., Christensen, L., Le, D. B. & Dahn, J. R. Alloy design for lithium-ion battery anodes. *J. Electrochem. Soc.* **154**, A849-A855 (2007).

8 Wang, J. *et al.* Structural evolution and pulverization of tin nanoparticles during lithiation-delithiation cycling. *J. Electrochem. Soc.* **161**, F3019-F3024 (2014).

9 Li, Q. *et al. In situ* TEM on the reversibility of nanosized Sn anodes during the electrochemical reaction. *Chem. Mater.* **26**, 4102-4108 (2014).

10 Nie, A. *et al.* Twin boundary-assisted lithium-ion transport. *Nano Lett.* **15**, 610-615 (2015).

11 Verbrugge, M. W., Qi, Y. , Baker, D. R. and Cheng, Y.-T. Diffusion□induced stress within core–shell structures and implications for robust electrode design and materials

selection in *Advances in Electrochemical Science and Engineering* (Eds. Alkire, R. C., Bartlett, P. N., Lipkowski, J.) Ch. 6, 193-226 (Wiley-VCH Verlag, Weinheim, 2015).

12 Zhao, K. *et al.* Concurrent reaction and plasticity during initial lithiation of crystalline silicon in lithium-ion batteries. *J. Electrochem. Soc.* **159**, A238-A243 (2012).

13 Chen, Q. & Sieradzki, K. Spontaneous evolution of bicontinuous nanostructures in dealloyed Li-based systems. *Nat. Mater.* **12**, 1102-1106 (2013).

14 Meduri, P., Clark, E., Dayalan, E., Sumanasekera, G. U. & Sunkara, M. K. Kinetically limited de-lithiation behavior of nanoscale tin-covered tin oxide nanowires. *Energ. Environ. Sci.* **4**, 1695-1699 (2011).

15 Sun, J. *et al.* Liquid-like pseudoelasticity of sub-10-nm crystalline silver particles. *Nat. Mater.* **13**, 1007 (2014).

16 Cao, K. *et al.* *In situ* TEM investigation on ultrafast reversible lithiation and delithiation cycling of Sn@C yolk-shell nanoparticles as anodes for lithium ion batteries. *Nano Energy* **40**, 187-194 (2017).
