# Supplementary material for: Strong stress-composition coupling in lithium alloy nanoparticles
Source: Nat Commun. 2019 Jul 31;10:3428. doi: 10.1038/s41467-019-11361-z (PMC6668403; doi:10.1038/s41467-019-11361-z)
Supplement: Supplementary file 8 — Description of Additional Supplementary Files [file 41467_2019_11361_MOESM8_ESM.docx]

Description of Additional Supplementary Files

**Supplementary Movie 1.**

A movie clip showing the lithiation dynamics and the accompanied phase evolution of a Sn-SnO_2_ core-shell nanoparticle (*a/b*=0.76). To take the electron diffractions in the course of lithiation, the electron beam density is reduced by half compared to the other movie clips. The movie is composed of alternative BF-TEM imaging and EDP parts recorded at 3 frames per a second. The fast forward rate varied to 32x, 16x or 2x between movie segments, to clearly show the phase and morphological evolution.

**Supplementary Movie 2.**

A movie clip showing the complete lithiation of a pristine Sn nanoparticle with minimal oxide shells (*a*/*b*=1). The lithiation dynamics exhibits no volume shrink or void formation, but continuous lithiation.

**Supplementary Movie 3.**

A movie clip showing the morphological evolution during stress-driven, spontaneous dealloying of lithiated Sn core in Sn-SnO_2_ core-shell nanoparticle (*a/b*=0.85). During the lithiation, the lithiated Sn core shrinks with Kirkendall void formation, while the shell thickness consistently increases.

**Supplementary Movie 4.**

A movie clip showing consistent dealloying during the lithiation process of a thin oxide-covered Sn nanoparticle (*a/b*=0.83) similar to that in Supplementary Movie 3. The particle initially expands in volume, followed by volume shrinkage and Kirkendall voiding.

**Supplementary Movie 5.**

A movie clip showing the continued lithiation of the once-pored core-shell nanoparticle (*a/b*=0.85) in Supplementary Movie 3. The particle core repeats lithiation and dealloying upon sufficient lithiation, until the shell partially tears from repeated mechanical interaction with the core.

**Supplementary Movie 6.**

A movie clip showing the lithiation of thick oxide shelled Sn nanoparticles (*a/b*=0.45 and 0.58). The particles undergo large mechanical constraint on the morphological evolution of the Sn cores during lithiation. Volume expansion is highly restricted and void formation occurs at earlier lithiation stage than those in the thin oxide shelled Sn nanoparticles shown in Supplementary Movies 1, 2 and 4.
